# Universal Sequence Replication, Reversible Polymerization and Early Functional Biopolymers: A Model for the Initiation of Prebiotic Sequence Evolution

**DOI:** 10.1371/journal.pone.0034166

**Published:** 2012-04-06

**Authors:** Sara Imari Walker, Martha A. Grover, Nicholas V. Hud

**Affiliations:** 1 NSF/NASA Center for Chemical Evolution, Georgia Institute of Technology, Atlanta, Georgia, United States of America; 2 School of Chemical and Biomolecular Engineering, Georgia Institute of Technology, Atlanta, Georgia, United States of America; 3 School of Chemistry and Biochemistry, Georgia Institute of Technology, Atlanta, Georgia, United States of America; University of South Florida College of Medicine, United States of America

## Abstract

Many models for the origin of life have focused on understanding how evolution can drive the refinement of a preexisting enzyme, such as the evolution of efficient replicase activity. Here we present a model for what was, arguably, an even earlier stage of chemical evolution, when polymer sequence diversity was generated and sustained before, and during, the onset of functional selection. The model includes regular environmental cycles (*e.g.* hydration-dehydration cycles) that drive polymers between times of replication and functional activity, which coincide with times of different monomer and polymer diffusivity. Template-directed replication of informational polymers, which takes place during the dehydration stage of each cycle, is considered to be sequence-independent. New sequences are generated by spontaneous polymer formation, and all sequences compete for a finite monomer resource that is recycled via reversible polymerization. Kinetic Monte Carlo simulations demonstrate that this proposed prebiotic scenario provides a robust mechanism for the exploration of sequence space. Introduction of a polymer sequence with monomer synthetase activity illustrates that functional sequences can become established in a preexisting pool of otherwise non-functional sequences. Functional selection does not dominate system dynamics and sequence diversity remains high, permitting the emergence and spread of more than one functional sequence. It is also observed that polymers spontaneously form clusters in simulations where polymers diffuse more slowly than monomers, a feature that is reminiscent of a previous proposal that the earliest stages of life could have been defined by the collective evolution of a system-wide cooperation of polymer aggregates. Overall, the results presented demonstrate the merits of considering plausible prebiotic polymer chemistries and environments that would have allowed for the rapid turnover of monomer resources and for regularly varying monomer/polymer diffusivities.

## Introduction

A key question in the origin of life is how the first biopolymers self-organized from simple chemical building blocks into increasingly complex systems with the capacity to cooperate and evolve [1]. Presently, most research that involves experimental models of early chemical evolution and function-based selection has necessarily utilized molecular systems that would have been possible only after considerable evolution of informational polymers [2]–[5]. Likewise, most theoretical models of early evolution have only considered how evolution could have produced the most efficient self-replicating entity after functional polymers had already emerged [6]–[10]. However, proposed scenarios for the chemical origins of life generally include, either explicitly or implicitly, a stage prior to functional polymer evolution when there was an initial buildup of informational polymers with random sequences – a stage that may have coincided with the selection of the first functional sequences [11], [12]. Exploring this early stage in chemical evolution is particularly difficult, being so obscured by the ensuing evolutionary history that even the identity of the first replicating material remains a subject of debate, with hypotheses ranging from RNA or some variety of proto-RNA [11], [13]–[16], to peptides [17], and even inorganic clay surfaces [18]. It also remains challenging to determine the environmental context from which the first informational polymers emerged, as illustrated by the wide variety of proposed sites for the origin of life, including (but not limited to): tidal shores [19], [20], deserts [21], hydrothermal vents [22], mineral surfaces [23]–[25], the eutectic phase of ice [26]–[28], deep underground [29], and even atmospheric aerosols [30].

Computer simulations offer a possible means to efficiently explore the potential merits of different scenarios for the emergence of polymer cooperativity and functionality [9], [10], [12], [31]–[37]. Here we explore simulations of polymer sequence and population evolution that incorporate several potentially important physical and chemical concepts of non-enzymatic replication and sequence evolution of prebiotic informational polymers, including: informational polymers with reversible backbone linkages [38]–[43], environmental cycling [21], [44]–[49], limited molecular diffusion [31], [35], [36], monomer recycling [16], [48]–[50], and template-directed synthesis without sequence restrictions [51]. Here we introduce the term Universal Sequence Replication (USR) to represent the possibility that prebiotic template-directed synthesis provided a means for the replication of polymers of a particular chemical structure (*i.e.* backbone and side chain structure), regardless of monomer sequence (such that replicative rate constants are, at least to first-order approximation, sequence-independent). USR should be distinguished from the more general framework of non-enzymatic template directed synthesis, which includes chemistries where certain sequences may have a replicative advantage; USR can be seen as a special case where the intrinsic fitness landscape is flat and no sequences possess an intrinsic replicative advantage.

Although the framework presented here shares some individual features with other models, their unification in the model presented provides a fresh perspective on a plausible scenario for environmentally-driven early emergence and evolution of informational polymers. Moreover, we diverge from most previous models in that we address the likely possibility that a nascent population of prebiotic informational polymers would have initially possessed no functional sequences (including no replicase activity). As such, we draw a distinction between the autonomous or mutually dependent “replicators” that have been explored in many previous models (*e.g.* quasispecies and hypercycle models [6]–[8]), and the implications of environmentally-driven USR that we explore here. This distinction highlights that the former class of models relies upon the self-replicating capacity of a particular polymer sequence or group of sequences, *i.e.* these models assume that functional polymers are already present in the extant population; whereas in the model explored here such a complex function has not yet emerged. Instead, USR posits that all polymers, regardless of monomer sequence, were on equal footing prior to the emergence of functionality.

Kinetic Monte Carlo simulations were used to explore the dynamics of populations of informational polymers that are formed by spontaneous polymerization, replicated by USR, and subject to hydrolysis in a diffusion-limited environment. The simulations reveal that a population of nonfunctional polymers is evolvable in the sense that new polymer sequences are continually introduced to a diverse extant population, and selection for new functional sequences can occur. The observed dynamics provide insights into a robust mechanism for the early exploration of sequence space, including how functional sequences might become established in an initially random sequence pool. Although USR imposes a flat replication fitness landscape, local feedback resulting from localized resource recycling and limited-diffusivity permit selection of functional sequences. A key result of our simulations is that functional selection does not dominate the system dynamics and species diversity remains high, where we define a species as a population of polymers with identical sequence. High species diversity during functional evolution permits the emergence and spread of more than one functional sequence, where nucleation of functional sequences may be temporally and/or spatially separated. Furthermore, functional sequences need not become permanently fixed within a population to beneficially impact population level dynamics. This rudimentary form of polymer cooperativity illustrates how evolution may have progressed at a level of the polymer pool before enzyme-based polymer replication or compartmentalization had emerged. The results presented suggest chemical features of candidate polymers and environments that might have initiated functional evolution of informational polymer populations in the origin of life.

## Methods

In constructing our model, we implemented a relatively small number of parameters to explore select chemical features of candidate prebiotic polymers and their physical setting, as well as the feedback between polymer and monomer populations. We first qualitatively outline the most salient features of the prebiotic scenario captured by our model, and then describe the specific details of how the model is numerically implemented.

### Model Description

In this section, we briefly describe the physicochemical features of our model, along with the associated adjustable model parameters. All model parameters (apart from enzymatic functional activity) are sequence-independent and are quoted in dimensionless units (see Supporting Information for a detailed description of model parameterization, Methods S1, including a table of parameters and values used in simulations, Table S1). Our primary motivation in choosing *all* kinetic rates to be sequence-independent was to focus on the role of the environment (*e.g.* cycling, diffusion) in driving system dynamics and functional selection (without biases introduced by differential kinetic rates). One exception is enzymatic functional activity, which is sequence-dependent in our model.

#### Regular environmental cycles

Environmental cycles would have occurred regularly on the prebiotic Earth (*e.g.* day-night, tidal, seasonal, hot-cold, freeze-thaw, hydration-dehydration, etc.). In the model presented here we appeal to hydration-dehydration cycles, such as those driven by tidal fluxes or day-night cycling, to provide an energetic source for the assembly of monomers into polymers, where physicochemical properties vary with the environmental phase. Polymerization via spontaneous assembly and USR via template-directed synthesis occur during the hot-dry conditions of the dehydrated phase. Polymer degradation and diffusion of monomers and polymers occur in the cool-wet conditions of the hydrated phase. Additionally, functional polymers (when present in the extant population) only exhibit catalytic activity during the hydrated phase, when cool-wet conditions promote the folding of a polymer into its active state.

#### Reversible polymerization

The model is based upon informational polymers with reversible backbone linkages [38]–[43]. During the hydrated phase, condensation polymers are subject to spontaneous degradation (hydrolysis), governed by the first-order rate constant 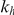. Monomers liberated via polymer hydrolysis are added back to the local monomer population, creating localized feedback between polymer and monomer concentrations.

#### Finite monomer concentration

The total number of monomeric units (*e.g.* nucleotides) in monomer and as polymeric residues is constant in our model system, with equal numbers of the two monomer species labeled 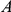 and 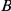. The choice to focus on closed mass systems was motivated by our goal of connecting to chemically realistic scenarios, where monomer concentration would have been naturally limited by available resources. For systems with a finite supply of resources, sustainable polymerization requires recycling through turnover of resources via polymer hydrolysis [16], [48]–[50]. An exception is the case where new monomers are generated by a functional polymer that acts as a monomer synthetase, where monomer production is still limited by the availability of precursor molecules, which are also a finite resource (see below).

#### Surface confinement and limited diffusion

Incomplete mixing, limited diffusion, and surface attachment have been shown to promote template-directed synthesis [51], [52] and to limit the deleterious effects of “parasites” (non-functional polymers) on selection of functional sequences [31], [35], [53]. In the present model, we therefore confine polymer and monomer movement to a surface, as might occur on a mineral with a thin film of water (or a more viscous solution) covering the surface, or other surface-binding phenomenon that permits the reversible association of monomers and polymers and limited movement in two dimensions. The reaction-surface is modeled as a square lattice (see below) where diffusion of monomers and polymers between neighboring sites is governed by the hopping rates 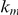 and 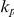, respectively, subject to the physical constraint condition 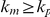. Diffusion only occurs during the hydrated phase of the cycle, when water activity is high. During the dehydrated phase, when the surface is dry, or the viscosity of the thin film is high, diffusion between lattice sites is considered to be negligible, with 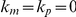.

#### Universal sequence replication (USR)

Recent studies have shown that altering the chemistry of nucleic acid backbone linkages (*e.g.* with a reversible linkage) [38], [39], [41], [43], or binding of template strands to a surface (*e.g.* reduced mobility compared to monomers) [51], allows for accurate template-directed synthesis for a wide range of sequences (*i.e.* approaching a form of USR). The effects of idealized USR are explored in our model, *i.e.* all polymers have the same intrinsic rate constant for replication, parameterized by the third-order rate constant 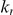. Despite the fact that all polymers share the same inherent replicative fitness, replication propensity is not the same for each polymer in each replication cycle, as the replication probability for a given polymer depends on both 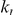 and the local monomer concentrations. Stochastic variation in the spatial distribution of free monomers results in spatiotemporally varying rates of polymer replication, creating a dynamic fitness landscape. As shown below, in the absence of functionality, this selective advantage is random, acting on local populations rather than individuals, with populations sequestering the most resources having an increased chance of survival, independent of their sequence distribution.

#### Spontaneous polymer assembly and exploration of sequence space

To simplify our model, we invoke spontaneous polymer assembly as the only means to generate novel sequences (*i.e.* we do not consider the effects of mutations during replication or of genetic recombination, which have been extensively studied elsewhere, *e.g.* see [6], [8], [31], [35], [37], [54], [55]). In this framework, polymer degradation allows continual exploration of sequence space as novel sequences are introduced via spontaneous assembly of free monomers. The rate of spontaneous assembly (to be contrasted with template-directed assembly) is governed by the second-order rate constant 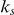, and local monomer concentrations. Throughout this work we set 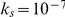, corresponding to a maximum global production rate of approximately 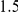 new sequences per dehydrated phase when all monomers are free (with lower rates when some monomers are sequestered in polymers). This value is intended to reflect a less efficient process of spontaneous polymerization as compared to template-directed synthesis.

#### Emergence of a functional polymer

A critical stage in the origin of life was the emergence of the first functional informational polymers [56]. We therefore explore with our model the case where a functional sequence is discovered by the random appearance of the sequence. Ma and coworkers have previously argued that in an RNA world, where polymer replication is accomplished without the need for enzymatic polymers (*i.e.* by some mechanism of USR [51], [57], [58]), that a nucleotide synthetase was the first enzyme to emerge [34]. A synthetase would have a clear advantage in an environment where local monomer concentration is the limiting factor for replication. We therefore explore a similar test case. In our simulations, the first polymer to emerge with a beneficial function is referred to as an 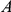
zyme, a polymer capable of synthesizing 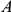 monomers from an additional but also finite resource, proto-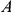 (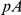). The catalytic activity of the 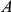
zyme is governed by the second-order rate constant 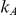 and local 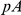 concentration. The 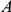
zyme thereby directly couples the functional dynamics to the local environment (*e.g.* local monomer abundance). Additionally, the emergence of a synthetase captures some properties of metabolic replicator models [34], [37], demonstrating a possible pathway between the pre-functional stage of replicating nonfunctional sequences and the subsequent stage of functional sequence optimization.

### Kinetic Monte Carlo Implementation

The simulations were implemented via a spatially-explicit hybrid kinetic Monte Carlo algorithm [59], [60]. Here we provide a brief summary of the technical details of our implementation. A more in depth discussion of the numerical implementation is provided in the Supporting Information (Methods S1).

#### Initial conditions and the reaction surface

The reaction surface is modeled on a 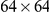 square lattice. Each lattice site represents a locally homogenous reaction domain, characterized by a freely interacting community of monomers and polymers (*i.e.* a locally well-mixed environment). The lattice size of 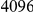 sites was chosen to be small enough for numerical tractability, yet large enough to allow spatial correlations to be observed for the range of kinetic and diffusive parameters under study (such that any spatial organization observed for the parameter ranges explored here is smaller than the lattice dimensions). We impose periodic boundaries to avoid edge effects. The total number of monomeric units (*e.g.* nucleotides) in monomer and as polymeric residues is constant, with equal numbers of the two monomer varieties labeled 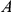 and 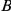. The initial conditions are homogeneous, with all mass in monomer: each of the 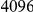 lattice sites is initialized with 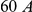 and 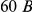 monomers, such that 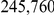 monomers each of species 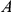 and 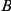 are evenly distributed over the reaction domain at the start of a simulation run. For functional runs in which the 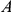
zyme appears, each site is also initialized with 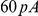 monomers, and with 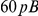 monomers for simulations where the 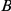
zyme also appears. No polymers are present at the start of a given simulation run (an exception is for functional runs, which start from an already established pool of random sequence polymers at quasi steady-state, see below).

#### Defining the polymer species pool

The number of possible polymer species, 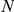, increases exponentially with polymer length: for a polymer of length 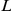 and two residue types there are 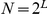 possible sequences. We chose to focus our study on polymer populations with a fixed length 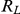, meant to approximate the dynamics of a population with a mean length of 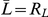 residues. For the simulations presented here, 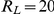. Our studies of the dynamics with other fixed polymer lengths, including 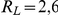 and 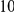, yielded qualitatively similar results. Although using shorter length sequences would have enhanced numerical efficiency, we used oligomers of length 20, as nucleic acids of this length are sufficiently long to adopt folded structures and, arguably, large enough to exhibit initial levels of catalytic activity [61]. Additionally, for 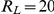 the sequence space is sufficiently vast that our simulations never explore all possible sequences within the timescales of our simulations. In other words, the sequence space is larger than what our system can dynamically explore in the space and timescales under study; satisfying the minimal requirement of a system with the potential for unlimited heredity and thus open-ended evolvability [1].

#### Assigning polymer composition

Our initial model implementation focused on polymers with fixed length 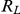 and any possible sequence diversity (*i.e.* any possible arrangement of 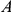 and 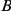 monomer residues could appear). However, sequences with a ratio 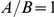 (*e.g.* composed of 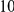 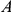 monomers and 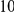 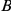 monomers for 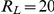) were an attractor for the dynamics under study, having the greatest access to available resources. Any deviations from the mean distribution, yielding a sequence population dominated by sequences with 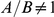, quickly returned to populations dominated by 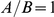 (as an example consider a pure 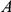-residue sequence which only has access to half the resources in the reactor pool and would quickly be outcompeted by other sequences containing some 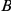 residues which had greater resource availability). Therefore, the simulations were set such that every sequence nucleated contained both 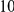 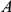 monomers and 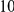 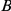 monomers. This greatly simplified polymer identification since each unique species need only be identified by a single ID or lineage number, 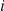 (a dramatic simplification over tracking the specific sequence structure of each unique lineage). This approximation reduced the size of the relevant sequence space from 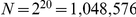 to 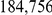 possibilities, but still retained our requirement that the relevant sequence space be much greater than what our dynamics could spatiotemporally explore within a given simulation run (see previous section), and allowed larger and longer statistical samplings.

#### Coupling of diffusive and kinetic events to environmental cycles

Our numerical implementation of the processes outlined in the previous section explicitly takes into account environmental cycling in order to accurately capture the dynamics of populations of condensation polymers with reversible linkages. The kinetic Monte Carlo implementation is therefore partitioned into two phases: a dehydrated phase, where all lattice sites are diffusively isolated (*i.e.* diffusion is turned off); and a hydrated phase, where lattice sites interact diffusively through polymer hopping events and monomer diffusion. Polymer assembly and replication occur only in the dehydrated phase, and polymer degradation occurs only in the hydrated phase. For simulations exploring the emergence of a functional sequence in the extant population, an additional kinetic process describing enzymatic catalysis is included in the hydrated phase.

#### The dehydrated phase

During the dehydrated phase, each lattice site 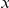 on the two-dimensional lattice is diffusively isolated and treated individually with the standard Gillespie algorithm [62]. The dehydrated phase supports the kinetic processes of spontaneous assembly and replication. The probabilities of reaction events are weighted by their relative reaction propensities. Spontaneous polymer assembly occurs with (second-order) reaction propensity
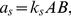
(1)and USR via template-directed assembly occurs with (third-order) reaction propensity
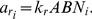
(2)Here 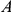, 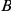, and 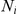 are the number of individual 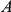 monomers, 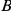 monomers, and polymers of lineage 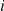 (the species ID number) at a specific site 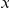. The total rate for polymer assembly and USR via template-directed assembly are governed by their respective rate constants, 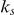 and 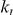, *and* the amount of available monomer resource. This dependence is essential to study how local resource availability affects the dynamics of polymer populations (for example, this feature leads to nontrivial spatial patterning, see Results). Since system dynamics are environmentally driven, a polymer may copy itself *at most* once per hydration/dehydration cycle. Therefore, after each template-directed replication event the total number of polymer templates available for further synthesis is decreased by one unit. In other words, we explicitly take into account that the template will not dissociate from the substrate until the next hydrated phase when dilution can drive dissociation. We note that cycling is often invoked as a mechanism for driving strand dissociation [63], however, it is not always explicitly included in model dynamics. We explicitly include cycling, along with its impacts on generational turnover, to explore the potential evolutionary impact on our model prebiotic system.

We note that USR via template-directed assembly is treated as a third-order process with a total rate dependent on resource availability through the nucleation term 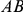 (in addition to the rate constant 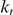 and availability of the template 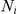 - see eq. 2). This relationship is meant to treat dimer formation as the rate limiting step for formation of full-length polymers, *i.e.* for the chemistries under investigation here, dimer association with a template is much stronger than monomer association. USR is treated as a one-step process for formation of full-length sequences, since the majority of polymers will form quickly once the first step of dimerization occurs. We therefore consider these approximations to be consistent with the physicochemical processes modeled, with the benefit that they lead to a simplified implementation of the numerical algorithm while still permitting resource dependence in the rates. The choice of kinetics for spontaneous polymer assembly has similar motivation. Dimers are expected to have stronger association to the reaction surface - *e.g.* mineral or clay - than monomers, thereby promoting polymerization once dimerization has occurred. The choice to implement resource dependence via a nucleation term 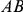 (rather than 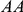 or 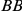) is consistent with our implementation of a reduced sequence space.

#### The hydrated phase

During the hydrated phase, monomers and polymers diffuse, and polymers may degrade or, if functionally active, perform catalysis. Individual lattice sites are diffusively coupled in the hydrated phase, and the dynamics are therefore modeled with a spatially-explicit hybrid kinetic Monte Carlo algorithm [59], [60]. All kinetic events are treated locally, occurring within an individual lattice site which is modeled as a locally homogeneous reaction domain, and only diffusive events occur between sites. A natural partition between rare and common events occurs due to the large separation in the population densities of monomer and polymer observed in our simulations. The simulations are therefore hybridized such that monomer site hopping is coarse-grained and treated via mass-action kinetics (see Supporting Information for more details, Methods S1). All other events in the hydrated phase are treated stochastically. We have verified that a full stochastic treatment (via a standard spatially-explicit Gillespie algorithm [64]) reproduces our hybrid results. Against the background of coarse-grained monomer diffusion, the rare polymer events of diffusion and degradation occur. The probabilities of (rare) reaction events are weighted by their relative reaction propensities. Polymer hydrolysis (degradation) occurs with reaction propensity
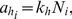
(3)and polymer diffusion (hopping between nearest-neighbor lattice sites) occurs with propensity
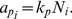
(4)Here hydrolysis is all or none, and degradation is therefore treated as a first-order one-step process which is stochastically determined. This approximation is made based on the implicit assumption that shorter polymer lengths are less stable, as might occur for cases where 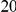
mers can maintain stable folded conformations whereas shorter length polymers cannot (*i.e.* we assume increasingly shorter length polymers become increasingly less stable to hydrolysis).

#### Data analysis

Data presented for quasi-steady state distribution averages, for explorations of both kinetic (*i.e.* replication/hydrolysis) and diffusive parameter space, are the combined result of time-averages over 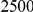 cycles and ensemble averages over a small statistical sampling of runs (averaged over 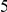 and 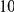 runs for kinetic and diffusive parameter space exploration, respectively). Small statistical samples are sufficient given the small spread in simulation values and the length of simulations with large time-sampling statistics. The quasi-steady state distribution is defined as the period when the ratio of polymer to monomer achieves an equilibrium value (with fluctuations due to stochastic effects). We used the term “quasi” here to indicate that the sequence population is not static, even at steady-state (see Results). Quasi-steady state distributions are calculated starting at 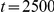 cycles (typically steady-state is achieved at 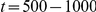 cycles depending on simulation parameters). Time averages were taken from 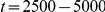 cycles. Data point error bars correspond to sample standard deviation on the mean time-averaged values. In comparing kinetic and diffusive processes for the results presented in this work it is important to note that the simulation dynamics are dependent on the overall rates of processes, which are dependent on both monomer and polymer abundances. The kinetic rate constants (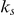, 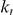, and 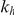) and the diffusive hopping rate constants (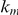 and 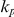) are useful in providing measures of the relative strengths of the processes under study, but must not be confused with the actual rates for the different kinetic and diffusive processes, which are dynamically determined by the ratios of monomer to polymer in a given simulation and their spatial distribution (as defined above, eqs. 1, 2, 3, and 4).

#### Simulating functionality

In simulations including the 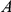
zyme, which catalyzes formation of 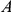 monomers from 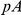 monomers, two additional processes are added to the hydrated phase, diffusion of 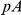 monomers and catalytic conversion of 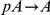. Diffusion of 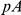 monomers, like diffusion of 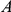 and 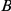 monomers, is treated via mass-action kinetics in the hybridized algorithm. Enzymatic catalysis by the 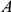
zyme is added to the rare events of the hydrated phase, with the probability of catalysis calculated from the reaction propensity
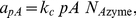
(5)where 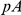 is the number of 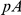 monomers on a local site 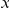, 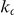 is the catalytic rate constant for conversion of 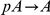 in the presence of the 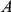
zyme, and 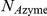 is the total number of 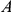
zymes on the local site 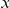. To illustrate the impact of the emergence of a functional sequence, data was saved at 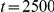 cycles for all details of a given simulation run. This data provided the initial starting distribution of monomers and polymer species for the functional runs. The choice of starting at 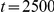 cycles is somewhat arbitrary given that the systemic dynamics in the quasi-steady state evolution are time-independent, but was chosen to be sufficiently late in the system evolution that a quasi-steady state had been established (*i.e.* the ratio of monomer to polymer was relatively constant). To this initial condition, 60 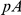 monomers were added to each site (to model a previously untapped resource in the environment) and a single polymer representing the 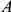
zyme sequence, or a nonfunctional sequence, was inserted on the lattice as a spontaneous assembly event. Results were averaged over twenty-five runs, each with a randomly chosen insertion point for the inoculated sequence. Selection of the inoculation site was weighted by the propensities for spontaneous assembly (*i.e.* inoculation was not completely random but determined by the resource distribution in the system as done for any other spontaneous assembly event). The simulations were permitted to run until the inoculated sequence (functional or nonfunctional) died out, or until the sequence had survived for 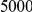 cycles. Lifetimes were averaged over survival times for the inoculated sequence lineage (defined as the duration of time where at least one individual of the inoculated sequence is still on the lattice) taken over twenty-five simulation runs. Population size averages, the average number of extant species, and exploration rate were averaged over the sequence lifetime. For example, a polymer that lives 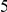 cycles is only averaged over 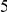 cycles, and therefore yields much higher variance in the data than nonfunctional simulations, which are averaged over entire populations of thousands of sequences, over thousands of cycles. Simulations including a 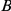
zyme, catalyzing 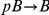, were inoculated in a similar manner with the initial simulation time taken at 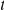 = 4000 cycles.

## Results

**Figure 1:**
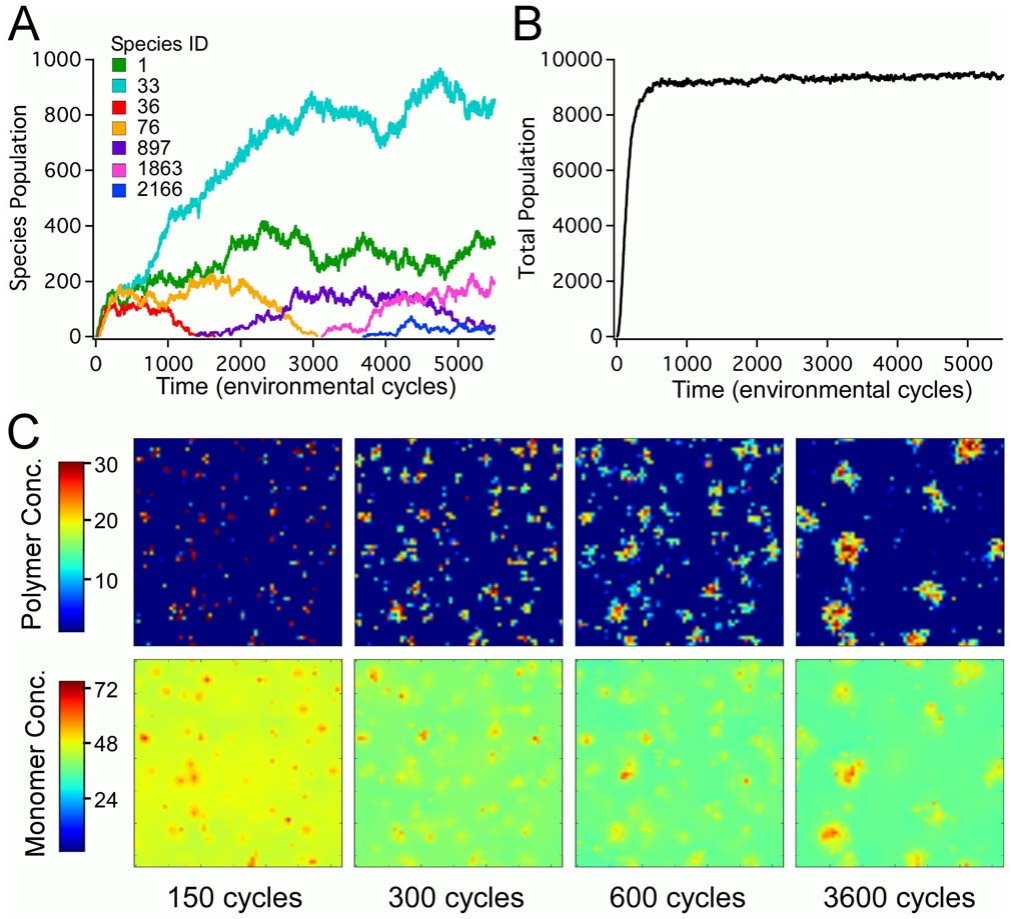
Sequence evolution of the polymer pool. A: Time evolution of the populations of seven specific sequences; B: Time evolution of the total polymer population; C: Spatial snapshots of the total polymer and monomer concentrations at four representative times. Species ID indicates the order of appearance of the first individual of a particular sequence in the polymer pool. The kinetic rate constants are 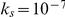, 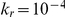, and 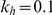, and polymer and monomer diffusivities are set as 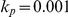 and 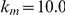 sites/cycle, respectively. Units of time are in number of cycles.

For each simulation run, the model system was initialized with 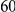 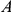 and 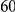 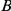 monomers at each of the 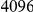 lattice sites, and no polymers. Starting from this homogeneous distribution, we tracked the spontaneous assembly, replication and spatial propagation of informational polymers over several thousand hydration-dehydration cycles. All stochastic events occur locally (*i.e.* within or between neighboring lattice sites); however, a global dynamic between local communities emerges due to diffusive contact. Stochasticity is observed to drive polymer population dynamics. In particular, for the parameter ranges investigated here, no system ever achieved a stationary steady-state population of polymers *with fixed sequence information*. Instead, the sequences represented in the polymer population continually change with time (Figure 1A). The *total population* of polymers is maintained once the system reaches equilibrium (Figure 1B), but the population is temporally varying with respect to the distribution of polymers among existing sequences and due to the appearance of new sequences – a state of dynamic kinetic stability (DKS) [65], [66]. Specifically, as individual polymers degrade, monomer recycling allows a quasi-steady state number of polymers to change in sequence distribution by providing resources for replication and spontaneous polymerization. Moreover, stochastic fluctuations in the number of individuals with a given sequence cause species to have a finite lifetime, while some new sequences that appear even after DKS has been achieved are observed to take hold and propagate in the population (Figure 1). We have verified that the quasi-steady states of the DKS observed in our simulations subsist for thousands of environmental cycles once an equilibrium distribution between monomer and polymer is established (running simulations upwards of 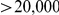 cycles). Stochasticity is also observed to drive dynamic pattern formation in the spatial distribution of polymers. A common feature of our simulations, for a wide range of parameter space, is the spontaneous appearance of polymer clusters. These localized high concentrations of polymers typically begin as a large number of small clusters that then coalesce into fewer, larger clusters over time (Figure 1C; time evolution movies of cluster formation are provided in Movie S1 and S2). Depending on the parameters of a given simulation, the space between clusters can be essentially devoid of polymers. An individual cluster is typically composed of multiple polymer sequences that mutually benefit from being part of a cluster. As will be shown below, cluster formation correlates with several important system characteristics, such as local monomer concentration, polymer lifetime, sequence diversity and functional sequence propagation.

### Exploring Replication and Hydrolysis Rate Parameter Space

**Figure 2:**
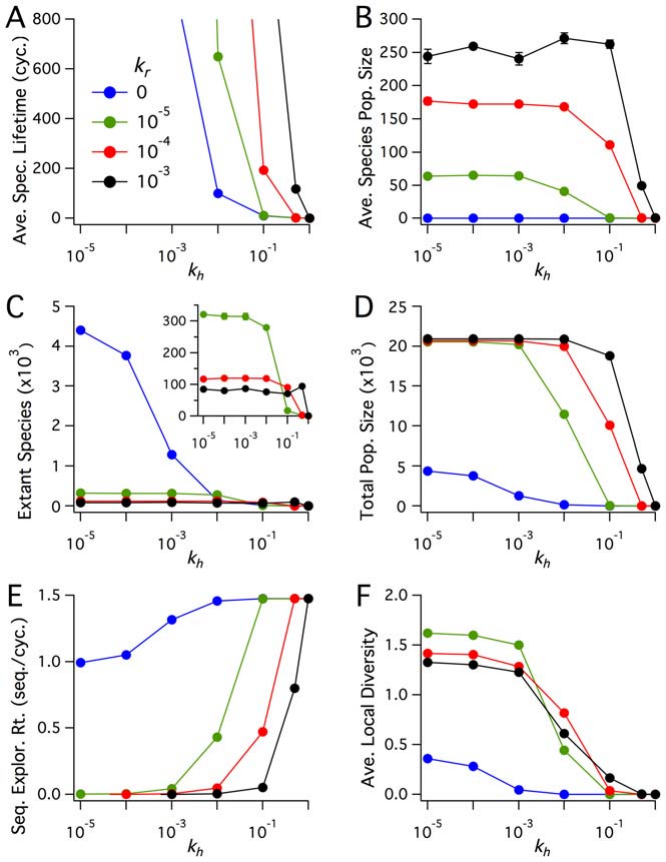
Exploring kinetic parameter space. Plots of quasi steady-state values of Average Species Lifetime (in units of number of cycles), Average Species Population, Extant Species, Total Population Size, Sequence Exploration Rate (in units of number of novel sequences generated per cycle), and Average Local Diversity. Time averages were taken from 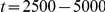 cycles, and each point is the ensemble average over five realizations. Error bars denote the sample standard deviation (most are smaller than symbols). The rate constant for spontaneous sequence nucleation is 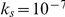, and the diffusion rate constants are 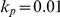 sites/cycle and 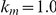 sites/cycle. The blue data set shows the reference case with 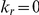, where no polymers replicate.

To explore system characteristics as a function of specific model parameters, we measured and compared ensemble averaged data for a number of system metrics collected for simulation runs in which one or two parameters were varied, with the remaining model parameters held constant. For the first set of simulations presented, the polymer replication and hydrolysis rate constants 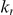 and 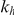, respectively, were varied while the rate constants for spontaneous polymer assembly and for monomer and polymer diffusion, 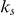, 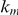 and 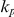, respectively, were held at fixed values. Since our aim is to investigate systemic features and evolutionary potential prior to the onset of functionality, no functional sequences exist in the systems presented in this section. As demonstrated by the data shown in Figure 2, the six system metrics of Average Sequence Lifetime, Average Species Population Size, Number of Extant Species, Total Polymer Population, Sequence Exploration Rate, and Average Local Diversity, each change to varying degrees in response to different values for 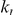 and 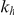.

Of the six metrics shown in Figure 2, the dependence of Average Species Lifetime on variations in 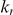 and 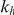 is, perhaps, most intuitive. A species is defined as a population of polymers sharing the same unique sequence of 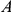 and 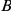 monomers, and the species lifetime is the number of contiguous cycles in which one or more copies of that sequence exists in the system. Due to the large number of possible sequences, it is assumed that a particular sequence will spontaneously appear in the system at most once over the timescales of interest. Figure 2A shows that Average Species Lifetime increases with replication rate constant 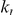, since higher polymer replication rates result in more *copies* of an extant sequence existing in the system. Species lifetime decreases with increases in the hydrolysis rate constant 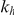, since all polymers have an increasing probability of spontaneous degradation. For the case of 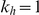, a polymer has a 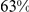 chance of degradation during each hydrated phase. Thus, most polymers do not survive into the next cycle, when they would have the opportunity to replicate. Consequently, for 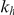 = 1 average polymer lifetime is 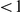 cycle for all values of 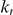 considered. In the case of no replication, when 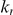 = 0, only one individual of any given sequence will ever exist in a simulation and therefore the Average Species Lifetime is determined only by the rate of polymer hydrolysis (*i.e.* by the average lifetime of a single polymer).

For 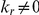 and 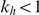, we observe that the ratio of 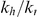 largely determines Average Species Lifetime (note: the ratio 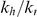 represents the ratio of *rate constants* and not the ratio of effective reaction rates which are dependent on local monomer and polymer densities). For 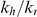 = 10, a lifetime 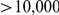 cycles is observed, while for 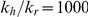, an average lifetime in the range of 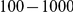 cycles is observed. For values of 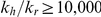, the Average Species Lifetime drops below 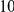 cycles. A distinction can be made in Figure 2A between systems with an Average Species Lifetime 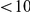 cycles and systems with an Average Species Lifetime of 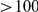 cycles. In the former, sequence lineage (*i.e.* species) propagation is minimal, whereas in the latter case, propagation of sequence lineages is robust. This result is also illustrated by other system metrics presented below.

Figure 2B shows the Average Species Population Size for extant species after DKS has been reached. Two regimes are clearly visible. In simulations with 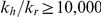, the Average Species Population Size is 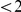. As with species lifetime, a high hydrolysis rate will limit the ability for polymers to replicate even for large values of 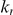, thereby limiting the propagation and copy number of any given species. For values of 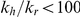 (with 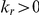), Average Species Population Size reaches a plateau value that is positively correlated with 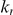. When 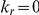, the case of no polymer replication, there can only be one copy of each sequence in the system regardless of hydrolysis rate, as is observed in Figure 2B.

Figure 2C provides a view of how the Number of Extant Species, defined as the average number of unique species present at any time after DKS has been reached, varies with 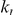 and 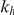. Again, for a sufficiently high hydrolysis rate (*i.e.* 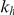 = 1), polymers generated in a given cycle are likely to degrade before having the opportunity to replicate, thus limiting the total number of polymers present in the system and thereby the Number of Extant Species. For 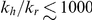, the Number of Extant Species reaches plateau values that decrease with increasing replication rate constant. This relationship is a direct result of competition for a finite supply of resources: larger replication rates lead to greater competition for the limited supply of resources, resulting in higher species extinction rates. In the case of no replication (*i.e.* 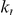 = 0), the Number of Extant Species is equal to the Total Polymer Population Size, *i.e.* the steady-state value is determined only by 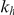, when all other parameters are held constant.

The Total Population Size of a system, shown in Figure 2D, is defined as the total number of polymers (regardless of sequence) present after DKS has been reached. Plots of this metric illustrate that for 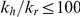, a plateau value of approximately 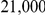 total polymers is reached. This value corresponds to roughly 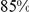 of monomers being sequestered in polymers, and 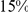 as free monomers. This upper limit on the number of monomers that can be incorporated into polymers is the result of an artificially imposed limitation on the simulation dynamics. Specifically, replication or spontaneous polymer formation cannot take place within a local environment that contains less than the number of monomers necessary to make a full-length polymer (*i.e.* twenty monomers, in our simulations with polymer lengths fixed at twenty). The combined systemic features shown in Figures 2B, 2C, and 2D show that for a wide range of hydrolysis and replication rates the same total number of polymers will be present in the system, but the population will be divided between a smaller number of unique sequences (or extant species) as the rate of replication is increased.

The metric of Sequence Exploration Rate is defined as the rate at which new sequences appear in a given system. Because new sequences arise solely by spontaneous formation, this metric provides a measure of the systemic ability to explore sequence space, an absolute necessity if a system is to evolve through the spontaneous appearance of polymers with functional activity. As shown in Figure 2E, for systems where 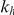 and 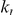 values give rise to a considerable percentage of free monomers in a state of DKS (*i.e.* small total polymer population sizes), the rate of sequence exploration is only limited by the parameter 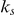, the rate constant for spontaneous polymer formation. As hydrolysis rates are decreased and replication rates increased, the number of free monomers available for spontaneous polymer formation decreases, thereby decreasing the Sequence Exploration Rate. For the case of 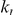 = 0, where polymers are generated only by spontaneous assembly, Sequence Exploration Rate remains high for a wide range of 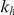 values, being only limited by the number of monomers made available via polymer hydrolysis. However, the special case of 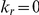 also corresponds to a system in which no sequences propagate through replication. Thus, for a system to evolve through the spontaneous discovery *and* propagation of a functional sequence, it is necessary that both the Sequence Exploration Rate be nonzero and that the Average Species Population Size be greater than one. For the system parameters explored here, values of 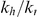 between 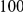 and 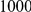 appear to be within a “sweet spot” of compromise between sequence exploration rates and the ability for a sequence to take hold in a system through replication.

Average Local Diversity is the final system-level metric shown in Figure 2. In contrast to the previous five metrics, Figure 2F describes the *spatial distribution* of sequences in the system. Sequence diversity was quantified using the Shannon entropy equation [67], and was calculated locally at each site on the lattice and averaged over the total number of lattice sites (see Supporting Information for additional mathematical details, Methods S1). Average Local Diversity therefore provides a statistical measure of the extent to which multiple sequences coexist on the same lattice site. As such, it provides a measure of diffusive mixing of populations (discussed below) and competition, whereby spatial regions with low local diversity result either from low diffusivity or high rates of local resource competition that result in fewer unique species. Unlike the previous plots, Average Local Diversity is strongly dependent on 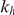, rather than the ratio 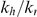. Low hydrolysis rates promote high local diversity, since extinction rates are low with high Average Species Lifetimes.

### Exploring Diffusive Parameter Space

We now present simulations designed to explore the effects of monomer and polymer diffusion rates on the system metrics defined above. Specifically, the polymer diffusive hopping rate, 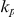, was varied within the range 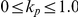, and the monomer diffusive hopping rate, 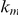, was varied within the range 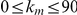. The simulation values investigated are subject to the condition 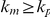, since polymers cannot diffuse faster than monomers. For the simulations presented, the remaining system parameters (*e.g.* the kinetic rate constants) were held fixed at values corresponding to intermediate metrics observed for the simulations presented in Figure 2 (*i.e.* 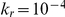; 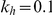; 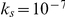). As in the previous section, our aim in this section is to investigate systemic features and evolutionary potential prior to the onset of functionality. Therefore, no functional sequences are present. Thus, all observed system characteristics presented in this section (including the onset of spatial patterning) arise in the absence of any polymeric catalytic activity (*i.e.* without functional sequences).

#### Spatial patterning

**Figure 3:**
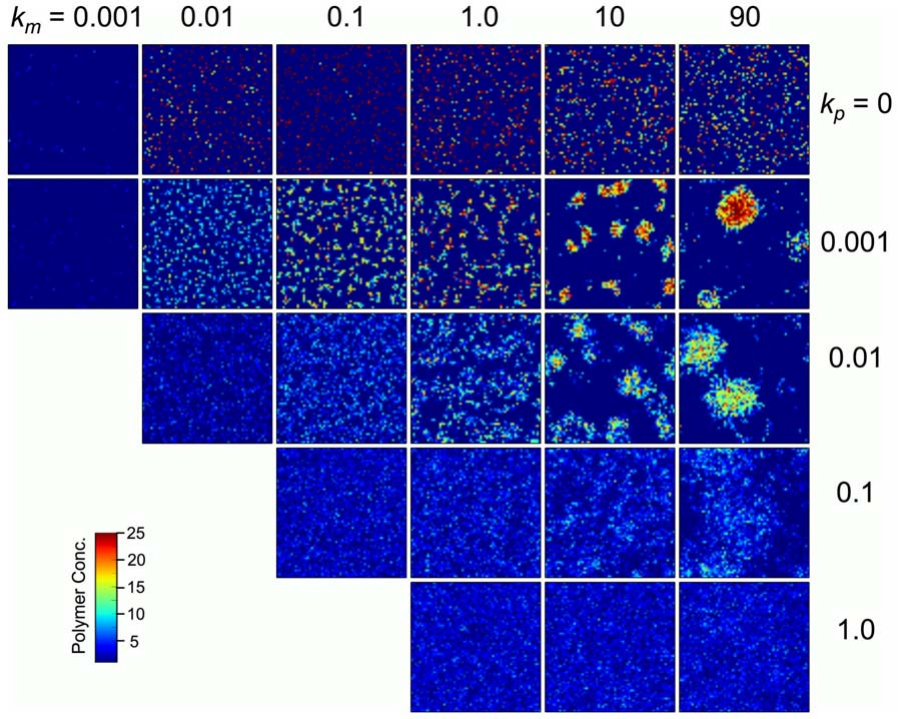
Spatial maps of polymer density. Each column of images corresponds to simulations run with a different value for monomer diffusivity 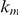 (in sites/cycle), and each row has a different value for polymer diffusivity 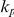 (in sites/cycle). All data shown are for kinetic rate constants 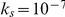, 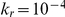, and 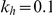. All maps correspond to 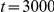 cycles. The color scale is in units of polymers/site. Simulations were only run for cases in which monomer diffusivity is greater than or equal to the polymer diffusivity.

Before discussing system metric results, it is instructive to consider the effects of varying 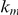 and 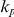 on the spatial distribution of polymers, as these diffusion-dependent distributions are important for understanding results for all other system metrics, as well as being interesting in their own right. As shown in Figure 3, simulations that have completed 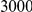 hydration-dehydration cycles exhibit polymer spatial organization that is highly dependent on the diffusivities 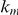 and 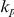, and their relative magnitude. All systems shown in Figure 3 had identical initial conditions – the system was initialized with a uniform distribution of monomers and no polymers at 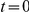. For monomer diffusive hopping rates 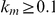, the total number of polymers in the system is fairly constant, with approximately 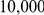 polymers on the lattice. However, large variations in the distribution of polymers are clearly visible. In simulations where monomers and polymers have low diffusivities (*e.g.* 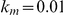 with 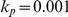), polymer species tend to stay spatially isolated and localized near their nucleation sites. Localized resource recycling sustains these small communities, with small polymer clusters being homogeneously distributed across the simulation lattice. As 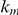 is increased from 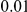 to 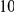, with 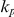 fixed at 0.001, polymer cluster size gradually increases until only one or two dominant clusters are observed after 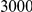 hydration-dehydration cycles. A similar trend of increasing cluster size is observed for simulations with a 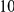-fold greater polymer diffusion rate, (*e.g.* 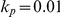), with larger, more diffuse clusters observed for greater polymer diffusion rates. Further increase in 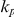 (*i.e.* 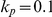 and 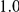 in Figure 3) leads to a loss of defined clusters, or clustering on a scale that is larger than the simulation lattice. In simulations with no polymer movement, 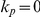, new sequences can only be introduced at a grid point by spontaneous polymerization, and clustering is not observed regardless of 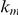 value. Additional spatial maps are provided in Supporting Information (Figures S1, S2, S3, S4, S5).

The clustering patterns observed in Figure 3 emerge due to the underlying stochasticity of the dynamics. As the first polymers nucleate and replicate, stochastic fluctuations in populations lead to inhomogeneities in polymer population densities. Populations with a slight initial excess of polymer grow by sequestering free monomers that diffuse to their local vicinity. Isolated polymers migrate toward these regions of concentrated resources or go extinct due to resource competition. Thus, clustering emerges as a result of an indirect form of cooperativity between replicating polymers, where early populations with higher polymer densities gain a fitness advantage. Polymers that exist within a cluster enjoy a local recycling dynamic in which the polymers of a cluster act as a reservoir of monomers: fresh monomer resources for replication become available upon polymer hydrolysis, where monomers are sequestered into polymers before these recycled resources have the opportunity to diffuse away from the cluster. This dynamic can occur because the overall rate of polymerization within a cluster is larger than the polymer diffusion rate, thereby resulting in localization of polymer populations (for polymer diffusivities with 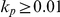 in Figure 3 strong clustering is not observed). Between the clusters, polymer density fluctuates near zero. These low population regions act as physical barriers to the transport of information between clusters, leading each aggregate to have a unique sequence population. In contrast, these polymer-depleted regions permit monomer transport, which, through stochastic fluctuations, allow growing clusters to acquire resources from shrinking clusters, even though the polymer clusters may not be in direct contact. The results shown in Figure 3 also illustrate that, for the parameters used in these simulations, polymer population growth is greatly inhibited if monomer diffusion is too slow. In particular, simulations carried out with 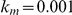 were almost devoid of polymers.

#### Diffusive dependence of system metrics

**Figure 4:**
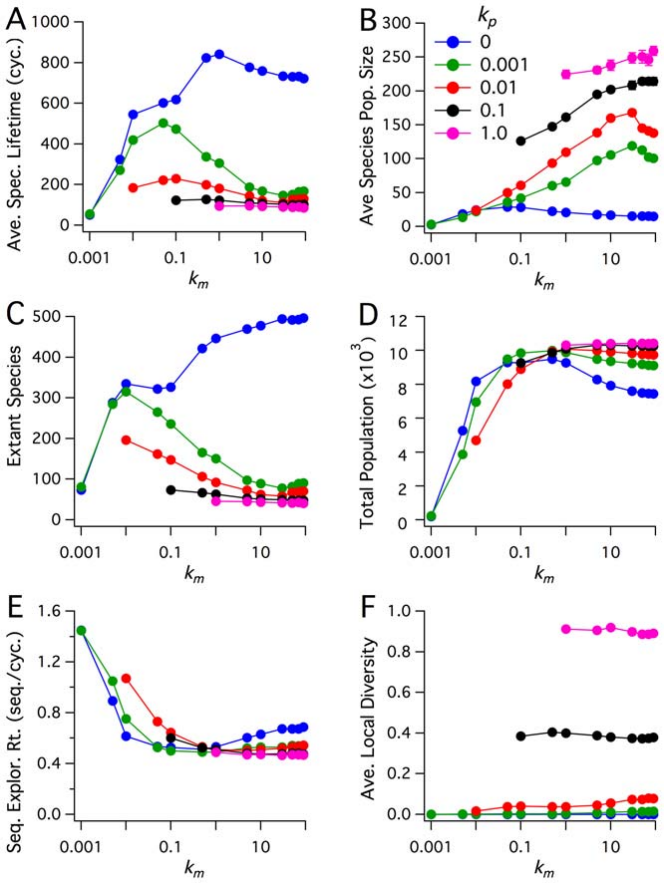
Exploring diffusive parameter space. Plots of quasi steady-state values of Average Species Lifetime (in units of number of cycles), Average Species Population, Extant Species, Total Population Size, Sequence Exploration Rate (in units of number of novel sequences generated per cycle), and Average Local Diversity. Time averages were taken from 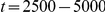 cycles, and each point is the ensemble average over ten realizations. Error bars denote the sample standard deviation (most are smaller than symbols). The kinetic rate constants are 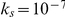, 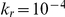, and 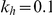. The blue data set shows the case where the 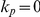, where polymers are completely immobile.

In Figure 4 the six system metrics are shown for the set of simulations in which 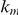 was varied from 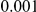 to 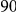, and 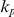 from 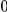 to 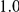. For nonzero polymer diffusion rates, Average Species Lifetime tends to decrease with monomer diffusion rates of 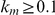 (Figure 4A). This trend is associated with the observed clustering at higher monomer diffusion rates. High diffusion rates allow for a small number of species to spread and sequester the majority of available resources, which causes high extinction rates for later-nucleating sequences. Thus, a species will, *on average*, have a shorter mean lifetime when a smaller number of species are able to dominate the sequestration of resources. The effect of diffusion on species growth is perhaps more easily appreciated by considering two other system metrics: Average Species Population Size and Number of Extant Species. Average Species Population Size (Figure 4B) shows a positive correlation between population size and polymer (and monomer) diffusion rates, with the average population size increasing with increased diffusivity. This result illustrates that increasing polymer and monomer diffusion rates leads to a decrease in the number of species that are able to dominate the acquisition of resources. Likewise, the Number of Extant Species (Figure 4C) shows that the mean number of extant species, like Average Species Lifetime, decreases with increasing polymer and monomer diffusion rates. In the special case of no polymer diffusion (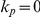), Average Species Lifetime and Number of Extant species increase, for the most part, with increasing monomer diffusion rates. This distinct trend is apparently due to increased monomer diffusion rates allowing for the replication of polymers before spontaneous hydrolysis, but without the ability for sequences to spread and “colonize” other regions of the surface, which also limits species population size. As noted above, the total number of polymers in a simulation after 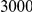 cycles was relatively independent of 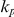, for 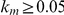 (Figure 4D).

In contrast to the results presented above for variations in hydrolysis and replication rates, we observe that the Sequence Exploration Rate (Figure 4E) is essentially independent of polymer diffusion rate and only modestly dependent on the monomer diffusion rate for the parameter ranges explored in Figures 3 and 4. Thus, when considering the ability for a system to evolve through sequence exploration, variations in monomer and polymer diffusivities are more likely to affect the capacity for survival of a newly nucleated sequence than the system's capacity to discover new sequences. For example, in the case of no polymer diffusion (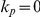), a sequence is not able to reap the benefits of spatial expansion, which permits population growth. On the other hand, a sequence that emerges in a system with high polymer mobility will experience strong competition, and may not take hold in the system. The ability for a *functional* sequence to overcome these pressures in explored below. Finally, the metric Average Local Diversity shows a unique response to changes in monomer and polymer diffusion rates. Like Average Species Population Size, Average Local Diversity increases with 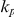. This positive correlation with polymer diffusion illustrates how more rapid polymer movement leads to more complete spatial mixing of polymer species. In contrast, unlike the five other system metrics, Average Local Diversity is apparently independent of 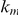, an observation that, taken with other system observations, demonstrates how variations in monomer diffusion rates can affect the spatial distribution of polymers without affecting the local spatial distribution of species diversity.

### Demonstrating the Emergence of Functionality

We now address the potential for an individual, catalytically active sequence to become established in a pre-existing pool of nonfunctional polymers. As introduced in The Model section, we chose for our test case the emergence of a polymer sequence (the 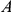
zyme) that catalyzes the production of 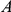 monomers from a previously untapped resource of proto-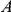 (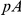) monomers. For these simulations, the system was initialized with 60 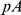 monomers at each lattice site (in addition to the 60 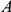 and 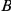 monomers), with a single 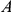
zyme sequence being introduced after the system of nonfunctional polymers had reached a state of DKS. For the results presented here, the 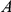
zyme was introduced at 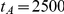 cycles. The catalytic rate constant of the 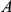
zyme was set sufficiently high that enzymatic activity would only be limited by access to 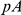 monomers. This criterion was satisfied by setting the catalytic rate of the 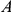
zyme such that one enzyme would be able to convert all 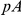 monomers within its lattice site to 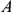 monomers within a single hydrated phase. Thus, the observed impact of the 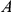
zyme sequence on the system does not depend on the catalytic activity of the 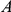
zyme, but instead on diffusive access to 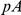 monomers, replication of the 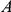
zyme sequence by USR, and the spread of this sequence by polymer diffusion.

#### Functional selection

**Figure 5:**
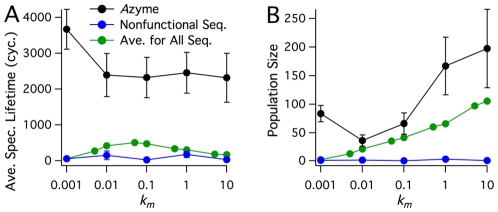
Selection for functional sequences. Plots illustrating the propagation of a functional 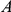
zyme, compared to nonfunctional sequences. A: Average Species Lifetime (in units of number of cycles), and B: Average Population Size. Each data point is the ensemble average over twenty-five runs, with error bars denoting the sample standard deviation. Kinetic rate constants are 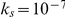, 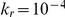, and 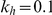, with a polymer diffusion rate constant of 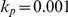 sites/cycle. The green points represent overall population statistics for realizations with no 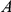
zyme (plotted in green in Figure 4). The black points represent statistics for a single functional 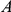
zyme. The blue points represent statistics for a nonfunctional sequence introduced at the same location and cycle as in the 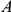
zyme simulations, except with no functionality.

Of the four system parameters explored above, the monomer hopping rate, 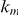, was selected for variation in a series of simulations in which the 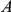
zyme sequence “spontaneously” appears. This parameter was chosen because diffusion of 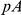 monomers into the vicinity of an 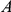
zyme, as well as the diffusion of newly synthesized 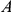 monomers away from a 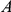
zyme, was expected to affect the ability for an 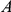
zyme to become established in a pre-existing population. In Figure 5, the Species Lifetime and Population Size of 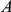
zyme lineages are shown for simulations in which 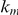 was varied from 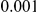 to 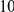. The polymer hopping rate, 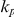, was fixed at 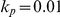, and all other parameters were the same as those used in the simulations presented in Figures 3 and 4. The selective advantage of the 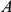
zyme over nonfunctional sequences is clear in these simulations, with the lifetime of an 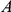
zyme sequence (shown in black) being 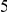 to 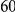 times as many cycles as the *average* lifetime of nonfunctional polymers (shown in green) in simulations with identical parameters, but without the appearance of an 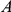
zyme. The population size of the 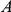
zyme, for all 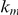 values explored, was approximately two times larger than the average population size of nonfunctional polymers in simulations where no 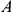
zyme appeared.

It is important to note that Species Lifetime and Population Size for simulations with nonfunctional polymers are weighted towards longer lifetimes and larger population sizes by sequences that appear early in the simulations. Early-time sequences are able to attain relatively large populations before the free monomer concentration begins to limit replication. Therefore, the average lifetimes and populations of early-time sequences are typically much greater in magnitude than those of nonfunctional sequences that emerge later, *e.g.* after DKS has been established. Thus, the observed enhanced species lifetime and population size of the late-appearing 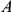
zyme is even more significant than it first appears relative to the green curve in Figure 5. To illustrate this point, simulations were carried out in which a nonfunctional polymer was introduced at the same cycle time and lattice site as the 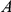
zyme (without the introduction of the 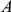
zyme). As shown by the blue curve in Figure 5A, the selective advantage of the 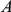
zyme sequence is, as expected, more dramatic when compared to the measured lifetimes and population sizes of the late-appearing nonfunctional sequence for all values of 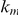.

The lifetime of an 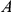
zyme sequence appears to be less dependent on 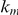 than the non-functional sequences, being of similar lifetime from 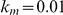 to 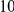. One exception is the considerable increase in 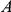
zyme lifetime observed in simulations with 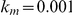. Under these conditions of very slow monomer diffusion, the lifetime of nonfunctional polymer sequences, even those that appear early in a simulation, are too short for any polymer lineages to become established and for a considerable population of any nonfunctional sequence to subsist. Thus, the results shown in Figure 5 also demonstrate that the 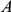
zyme is able to survive in an environment that cannot sustain nonfunctional polymer populations. In other words, the 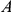
zyme, by significantly enhancing its own survival, can become the first sustainable extant sequence in a highly dynamic and previously unsustainable environment.

#### System-level benefits of a functional sequence

**Figure 6:**
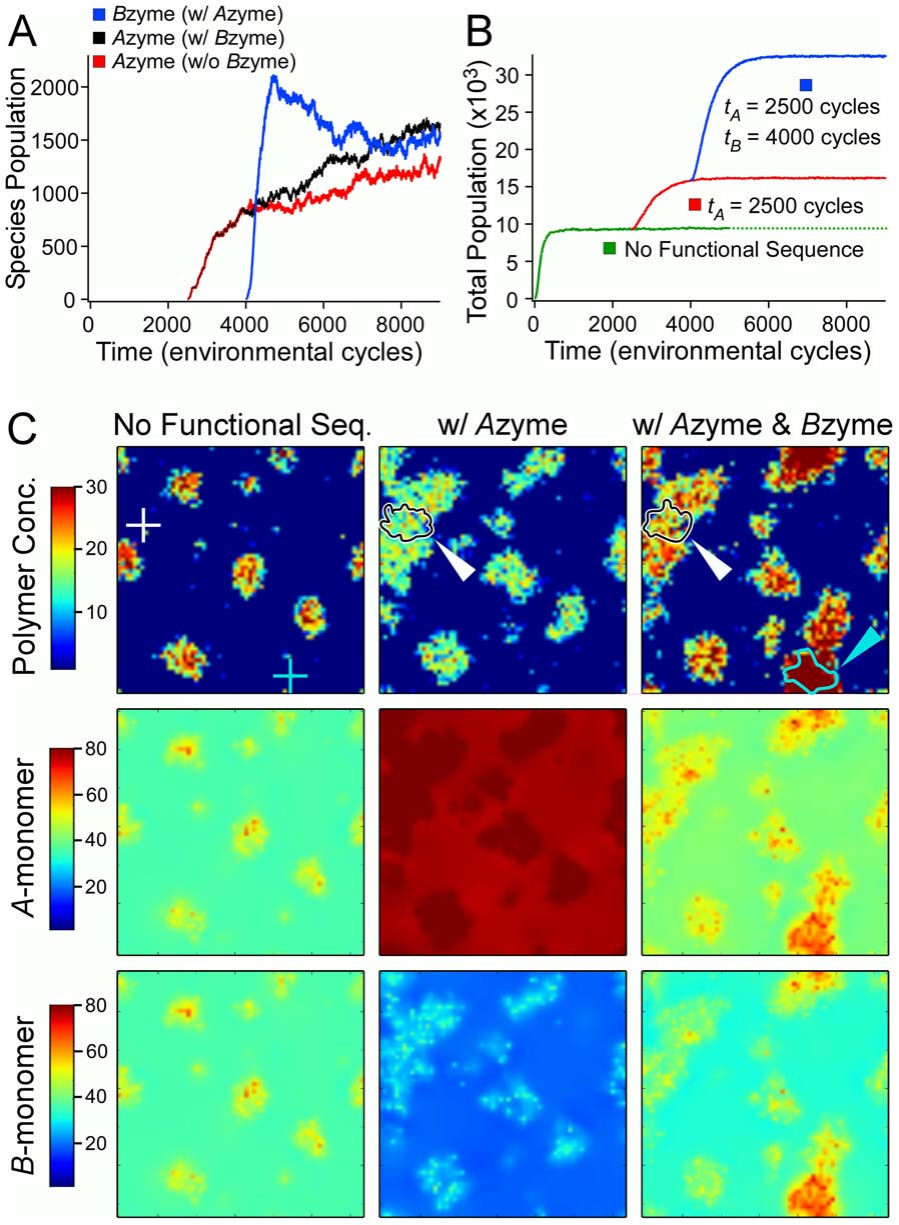
Spatial distribution maps for no functional species, one functional species, and two functional species. The three scenarios shown are all identical up to 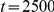 cycles, at which time the system has achieved a quasi-steady state distribution. In the first scenario, no functional sequences appear. In the second scenario, a functional 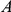
zyme appears at 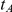 = 2500. In the third scenario, the same functional 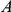
zyme appears at 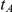 = 2500, and a functional 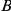
zyme also appears at 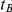 = 4000. A: Time evolution of the Species Populations of the 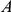
zyme and 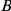
zyme. The units of time are in number of cycles. The red curve corresponds to the second scenario, having only the 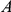
zyme, while the black and blue curves correspond to the third scenario with both enzymes emerging. B: The time evolution of the Total Polymer Population for the three scenarios. C: The spatial distribution of the polymer (total) and monomer concentrations, at 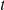 = 5000 cycles. White arrow indicates contour containing 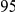
% of 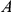
zyme polymers, cyan arrow indicates contour containing 95% of 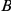
zyme polymers. Kinetic rate constants are 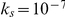, 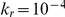, and 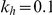, and diffusive rate constants are 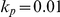 and 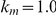 sites/cycle.

The population of the 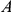
zyme is plotted in Figure 6A as a function of time (in units of cycles) for a simulation with 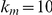. A comparison of this plot with those of simulations with only nonfunctional sequences reveals that the population growth of this sequence is faster and to a similar level of the most successful early-appearance polymers (*e.g.* Sequence ID 33 in Figure 1A). The effect of 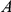
zyme appearance on the overall system can be appreciated by comparing the total polymer population after the appearance of the 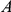
zyme to the steady-state polymer population that is maintained by nonfunctional sequences (Figure 6B). In these simulations, the 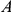
zyme becomes well established in the population, but its population growth represents only about 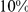 of the total increase in polymer population. Thus, most of the newly formed 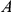 monomers are used to generate new sequences and to replicate existing nonfunctional sequences. While this result might, at first, be considered deleterious for the 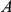
zyme and a waste of resources on “parasitic” nonfunctional sequences, it must be realized that the allocation of resources to other sequences is positive at a system level, as the capacity to search sequence space and to propagate other functional sequences is enhanced.

A comparison of 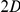 spatial maps of polymer and monomer densities for systems with and without the introduction of the 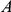
zyme sequence further illustrates the effects of a functional 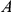
zyme on a system of otherwise nonfunctional polymers (Figure 6C). The location of the initial appearance of the 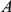
zyme sequence is essentially devoid of polymers at 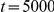 cycles in the simulation containing only nonfunctional polymers. In contrast, introduction of the single 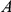
zyme sequence at 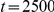 cycles results in a substantial change in the local and global polymer distribution. A cluster grows around the site of 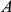
zyme introduction, which eventually merges with nearby clusters of nonfunctional sequences. Within this larger cluster the 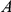
zyme coexists with nonfunctional sequences that benefit from the temporal increase in local free 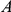-monomer concentration. Dramatic differences in 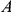-monomer and 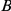-monomer densities are also observed across the simulation surface (Figure 6C). The 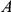 monomer is no longer a limiting factor in polymer production, and eventually increases to higher than pre-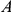
zyme levels at every point on the simulation domain. In contrast, the 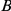 monomer becomes a more strongly limiting reagent to polymer production, with 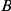-monomer levels dropping well below those of the pre-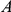
zyme levels across the simulation surface as more resources are consumed by the larger polymer population.

#### Functional cooperation over time and space

Having demonstrated that a single functional sequence can become established within a system of nonfunctional polymers, we next investigated the effects of adding a second functional sequence that acts as a catalyst for the conversion of a proto-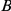 monomer (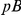) to 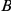 monomer. As shown in Figure 6A, in simulations where 60 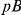 monomers were initially present at each lattice site, the appearance of a single 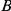
zyme sequence at 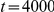 cycles (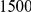 cycles after the appearance of the 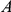
zyme) quickly results in a burst of 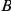
zyme population growth. As was observed for the 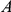
zyme, the growth in 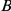
zyme population represents only a fraction of the total increase in the global polymer population size (Figure 6B). A plot of polymer density 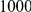 cycles after the appearance of the 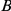
zyme shows the growth of a large cluster with the 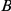
zyme population at its center. Other polymer clusters on the surface show substantial growth as a result of the appearance of the 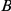
zyme.

An important feature of the system explored here is that the selective pressure for a functional polymer can be transient in time and space. Each simulation run shows slightly different dynamical evolution after the appearance of a functional polymer. 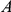
zyme and 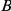
zyme population plots and 2 *D* density plots for a second example are provided in the Supporting Information (Figure S6) for a simulation with 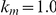 (as compared to 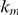 = 10 for the simulations shown in Figure 6). For this simulation where only the 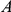
zyme appears at 2500 cycles (the 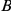
zyme does not appear), the 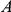
zyme sequence goes extinct within the next 6000 cycles. In contrast, when the 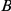
zyme is nucleated near the center of 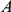
zyme activity, survival of both functional sequences is enhanced. We note that extinction of the 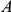
zyme, in the absence of 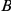
zyme appearance, does not terminate system evolution. Figure S6B illustrates that the pool of polymers benefited from the transient activity of the 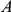
zyme. Furthermore, the 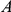
zyme had nearly exhausted its benefit to the system at the time of extinction, having had converted nearly all 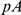 to 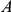 monomers. However, a stable cluster emerged where the 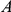
zyme nucleated (Figure S6C), leading to localized enhancement of polymer density and number of extant species. This result demonstrates that the 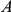
zyme (or any other functional sequence) is not required to live indefinitely in order to have a positive impact on a system undergoing continuous rounds of USR. Moreover, once the 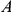-monomer is no longer a limiting reagent, it would be a distinct disadvantage for the 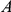
zyme population to remain high: for continued system-level evolution, it is more advantageous for the monomers in the 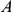
zyme sequences to be recycled into polymers with functions that are needed at later times.

## Discussion

The physical environment and the molecules available on the prebiotic Earth would have placed tremendous constraints on *any* mechanism that led to the evolution of informational polymers with functional activity. Among these constraints would have been limited resources and finite polymer stability. With these particular constraints in mind, we used a kinetic Monte Carlo simulation to explore the emergence of functional polymers when only nonfunctional polymers existed that were all replicated with equal probability, regardless of sequence. The model utilizes a minimal set of adjustable parameters in order to explore the effects of those parameters considered most relevant to this putative early stage of prebiotic evolution. The results of our simulations have revealed the possible existence of regions in physiochemical parameter space that could have supported the constant exploration of sequence space, as well as the selection of functional sequences.

Our simulations demonstrate how variations in polymer hydrolysis and replication rates affect the ability for a pool of informational polymers to explore sequence space, even after an equilibrium population of polymers has been established. As expected, systems with faster polymer hydrolysis rates allow more rapid exploration of sequence space, due to more rapid turnover of resources. However, for a system to take advantage of functional sequences that appear spontaneously, the polymer replication rate must be sufficient to counter the deleterious effect of rapid polymer degradation, otherwise information propagation is not sustainable and no polymer lineages become established. Conversely, low polymer degradation rates can cause extant (and nonfunctional) polymers to unproductively retain monomer resources, severely limiting the rate of sequence space exploration. For the model parameters explored here, we have found that a region of compromise exists for a range of replication and hydrolysis rates that allows a nonzero rate of sequence exploration and a nonzero probability that new sequences become established in the system.

To demonstrate functional sequence selection we focused on the appearance and propagation of monomer synthetases. For this particular functionality, the appearance of the 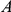
zyme and 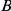
zyme in the extant population at different points in space and time illustrates a rudimentary form of cooperativity between two functional lineages that may be spatially and/or temporally separated. We argue that such a scenario for functional sequence emergence and early sequence cooperation is more plausible for the earliest stages of prebiotic evolution than scenarios that require the first functional polymer to have been a much more complicated enzyme (*i.e.* a processive polymer replicase [54]), or for the diverse members of a set of enzymes to emerge *de novo* at the same point in space and time [68]. As the system evolved, there is no reason not to expect that continued system dynamics would eventually permit more complicated catalytic functionalities to be selected and optimized, perhaps even culminating in the eventual appearance of polymerases [69] and ligases [70].

Many origin of life researchers consider polymer compartmentalization, such as nucleic acid encapsulation in lipid vesicles [71], to be a prerequisite for evolution. It is certainly true that there must exist a means for the co-localization of functional polymers with the “fruits of their labor”. However, limited diffusion (or incomplete mixing) has been shown to provide a possible alternative to encapsulation [9], [32], [36], at least in the earliest stages of prebiotic evolution. As shown here, system-level metrics, such as Average Species Size and Exploration of Sequence Space, depend on the rates of molecule movement between lattice sites, illustrating that limited movement, representing a realm between stringent compartmentalization and homogenous mixing, could have been beneficial in the early stages of informational polymer growth and evolution. Furthermore, even without employing explicit compartmentalization, nearly all diffusion-limited regimes explored here support stable and diverse populations of extant sequences, and some promote the dynamic emergence of spatial aggregates, which appear even in the absence of any functional activity. In particular, our simulations illustrate how nonfunctional informational polymers can play a positive role by contributing to a local recycling dynamic that sustains extant polymer populations. As one consequence, in all diffusive regimes explored, the unit of selection (or survival) is not the individual polymer, but local populations of polymers dynamically coupled through resource recycling. These populations act both cooperatively as competitive aggregates and individually through single polymer replication/degradation/diffusion events, and through functional selection of active sequences. Thus, the results presented here reinforce previous assertions that physical compartmentalization is not necessary for prebiotic evolution [9], [36].

The effects of parasites are not absent in our models. For example, in simulations where 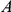
zyme and 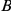
zyme sequences emerge, the majority of monomer resources created by these functional sequences become incorporated into nonfunctional sequences. The observed dynamics are similar to that of the nonfunctional parasites in the model of Könnyű and Czárán [37]. However, in contrast to the dynamics observed in their metabolic autonomous replicator model, where parasites are tolerated by functional sequences but play no active role, parasites in the prebiotic scenario presented here are not completely deleterious. When a synthetase first emerges nonfunctional sequences may be beneficial by providing a localized enhancement of resources in an existing polymer cluster that locally retains the new monomer resources (within recyclable polymers) as the synthetase sequence gradually increases in number. At the very least, nonfunctional sequences that take up monomers generated by the synthetase can later provide raw materials for the continued search of sequence space, thereby increasing the survivability and evolvability of the system as a whole.

In conclusion, we have shown that system-level phenomena can emerge from a pool of monomers and replicating polymers that are governed by a small number of meaningful chemical and physical parameters. Moreover, we have shown that in a system of polymers where there is no intrinsic sequence-specific replicative advantage, evolution can still take place. At the rudimentary level – before the appearance of functional sequences – environmental cycling, limited diffusivity, and resource limitation leads to the spontaneous self-organization of polymers into spatial aggregates. Even in the idealized case of universal sequence replication, a dynamic fitness landscape spontaneously appears. When functional sequences eventually appear, they can become established amid the nonfunctional polymers, and enhance the ability for other sequences to evolve across space and time. Taken together, these results allow qualitative predictions about the chemistries and environments that would have facilitated prebiotic sequence evolution. Specifically, our model suggests that the optimal conditions for the earliest stage of abiotic chemical evolution, prior to the onset of functional evolution, would *not* have been those that promote stringent maintenance of sequence information *per se*, which is considered optimum for most autonomous replicator models [54], including that of Eigen [6], [8]. On the contrary, the optimum conditions for early informational polymer evolution would have allowed the spontaneous appearance of completely new sequences, and for the existence of new sequences to be just long enough for functional sequences to gain a local selective advantage during subsequent cycles of replication. Future investigations of the earliest replicating polymers of life should therefore place more emphasis on polymer chemistries and environments that allow for rapid turnover of resources and time-varying diffusivities for monomers and polymers. Finally, in light of the possibility that compartmentalization appeared after the onset of functional polymer evolution, the results presented here support a model for the early stages of biopolymer evolution that are dramatically different from that governed by a strictly Darwinian process. That is, these early stages could have been defined by the collective evolution of a system-wide cooperation of polymer aggregates. The same general characteristics have been proposed by Woese for the earliest biological systems, but for reasons based on bioinformatics analyses of extant organisms [72], [73].

## Supporting Information

Figure S1
**Spatial maps for** 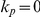 **sites/cycle.** Spatial maps of polymer density (top row), 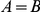 monomer density (middle), and local diversity (bottom) for 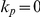 sites/cycle, with monomer diffusivity increasing from left to right. Polymers do not diffuse and as such are indefinitely stuck on their nucleation site. Snapshots are taken at 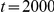 cycles. The kinetic rate constants are 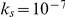, 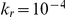, and 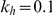.(TIF)

Figure S2
**Spatial maps for** 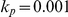 **sites/cycle.** Spatial maps of polymer density (top row), 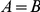 monomer density (second row), local diversity (third row), and similarity index (bottom row) for 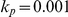 sites/cycle, with monomer diffusivity increasing from left to right. Snapshots are taken at 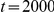 cycles. The kinetic rate constants are 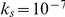, 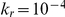, and 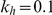.(TIF)

Figure S3
**Spatial maps for** 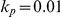 **sites/cycle.** Spatial maps of polymer density (top row), 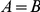 monomer density (second row), local diversity (third row), and similarity index (bottom row) for 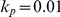 sites/cycle, with monomer diffusivity increasing from left to right. Snapshots are taken at 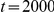 cycles. The kinetic rate constants are 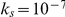, 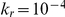, and 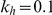.(TIF)

Figure S4
**Spatial maps for** 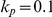 **sites/cycle.** Spatial maps of polymer density (top row), 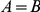 monomer density (second row), local diversity (third row), and similarity index (bottom row) for 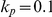 sites/cycle, with monomer diffusivity increasing from left to right. Snapshots are taken at 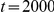 cycles. The kinetic rate constants are 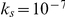, 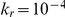, and 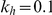.(TIF)

Figure S5
**Spatial maps for** 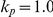 **sites/cycle.** Spatial maps of polymer density (top), 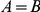 monomer density (second row), local diversity (third row), and similarity index (bottom row) for 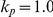 sites/cycle, with monomer diffusivity increasing from left to right. Snapshots are taken at 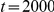 cycles. The kinetic rate constants are 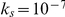, 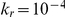, and 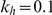.(TIF)

Figure S6
**Spatial distribution maps for no functional species, one functional species, and two functional species.** The three scenarios shown are all identical up to 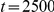 cycles, at which time the system has achieved a quasi-steady state distribution. In the first scenario, no functional sequences appear. In the second scenario, a functional 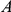
zyme appears at 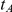 = 2500. In the third scenario, the same functional 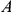
zyme appears at 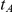 = 2500 cycles, and the functional 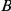
zyme appears at 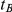 = 4000 cycles. In Panel A, the time evolution of the Species Populations of the 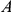
zyme and 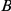
zyme is shown. The red curve corresponds to the second scenario, having only the 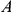
zyme, while the black and blue curves correspond to the third scenario with both enzymes emerging. Panel B shows the time evolution of the Total Polymer Population for the three scenarios. Panel C illustrates the spatial distribution of the polymer (total) and monomer concentrations, at 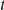 = 5000 cycles. Kinetic rates are 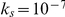, 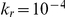, and 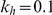, and diffusive rates of 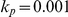 and 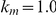 sites/cycle.(TIF)

Movie S1
**Spatial distribution maps for no functional species, one functional species, and two functional species.** The three scenarios shown are all identical up to 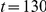 cycles. In the first scenario, no functional sequences appear. In the second scenario, a functional 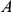
zyme appears at 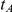 = 130 cycles. In the third scenario, the same functional 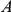
zyme appears at 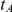 = 130 cycles, and the functional 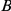
zyme appears at 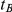 = 209 cycles. The spatial distribution of the polymer (total) and monomer concentrations are shown from 0 up to 1000 cycles. The kinetic rate constants are 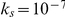, 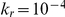, and 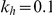, and diffusive rates of 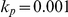 and 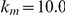 sites/cycle. The scale for polymer density spans from 0 (blue) to 30 (red). The scale for monomer density spans from 0 (blue) to 100 (red). The scale for similarity index spans from 0 (blue) to 1 (red).(M4V)

Movie S2
**Spatial distribution maps for two functional species.** The functional 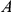
zyme appears at 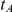 = 130 cycles, and the functional 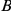
zyme at 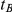 = 209 cycles. The spatial distribution of the polymer (total), monomer, and proto-monomer concentrations are shown from 0 up to 1000 cycles. The kinetic rate constants are 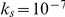, 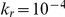, and 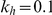, and diffusive rate constants are 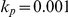 and 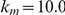 sites/cycle. The scale for polymer density spans from 0 (blue) to 30 (red). The scale for monomer density spans from 0 (blue) to 100 (red). The scale for proto-monomer density spans from 0 (blue) to 60 (red). The scale for similarity index spans from 0 (blue) to 1 (red).(M4V)

Methods S1 (PDF)

Table S1 **Parameters and values used in simulations.**
(PDF)
