# Supplementary figures and images for: Universal Sequence Replication, Reversible Polymerization and Early Functional Biopolymers: A Model for the Initiation of Prebiotic Sequence Evolution
Source: PLoS One. 2012 Apr 6;7(4):e34166. doi: 10.1371/journal.pone.0034166 (PMC3320909; doi:10.1371/journal.pone.0034166)

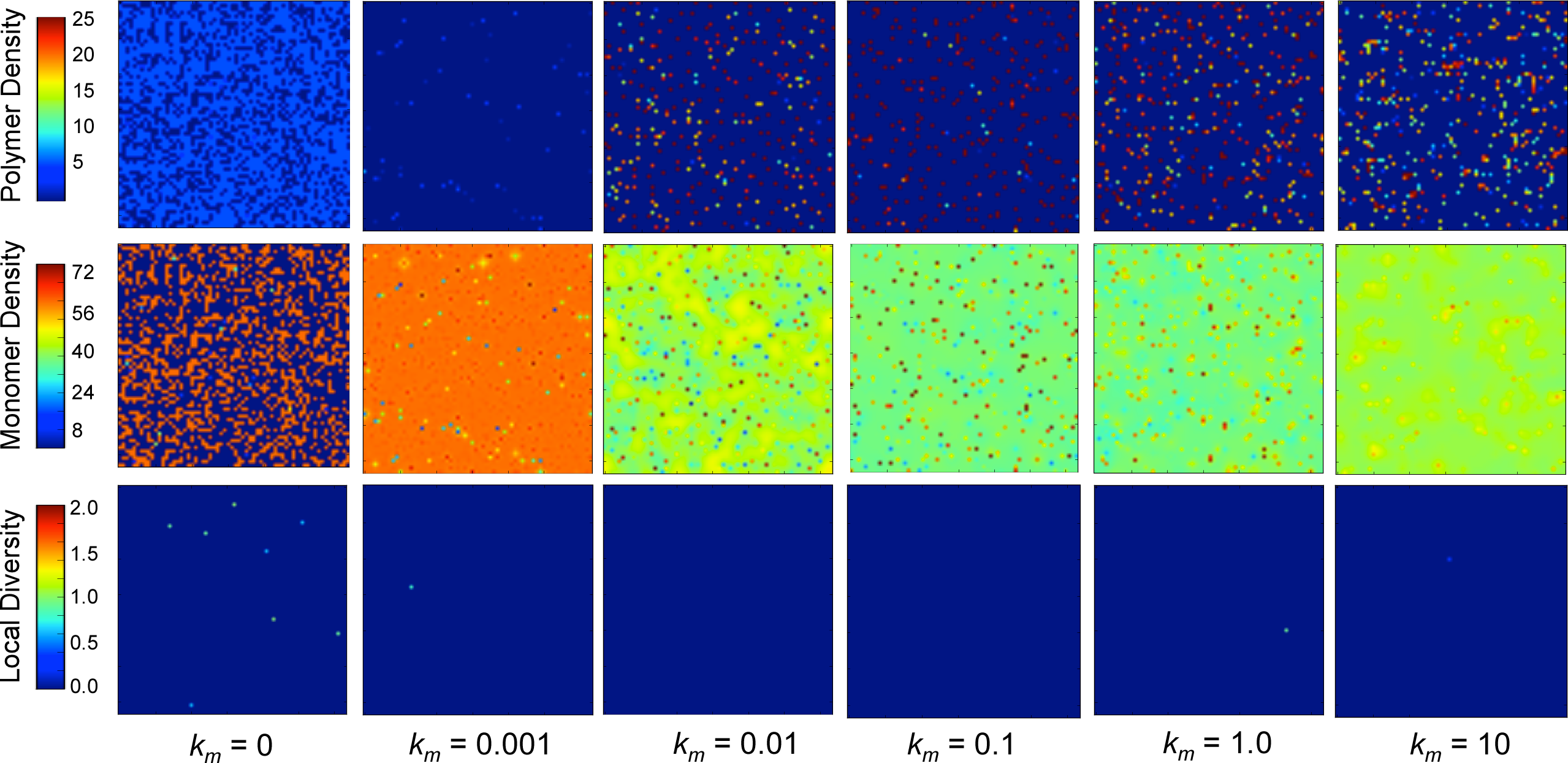

Supplement: Figure S1 — Spatial maps for sites/cycle. Spatial maps of polymer density (top row), monomer density (middle), and local diversity (bottom) for sites/cycle, with monomer diffusivity increasing from left to right. Polymers do not diffuse and as such are indefinitely stuck on their nucleation site. Snapshots are taken at cycles. The kinetic rate constants are , , and . (TIF) [file pone.0034166.s001.tif]

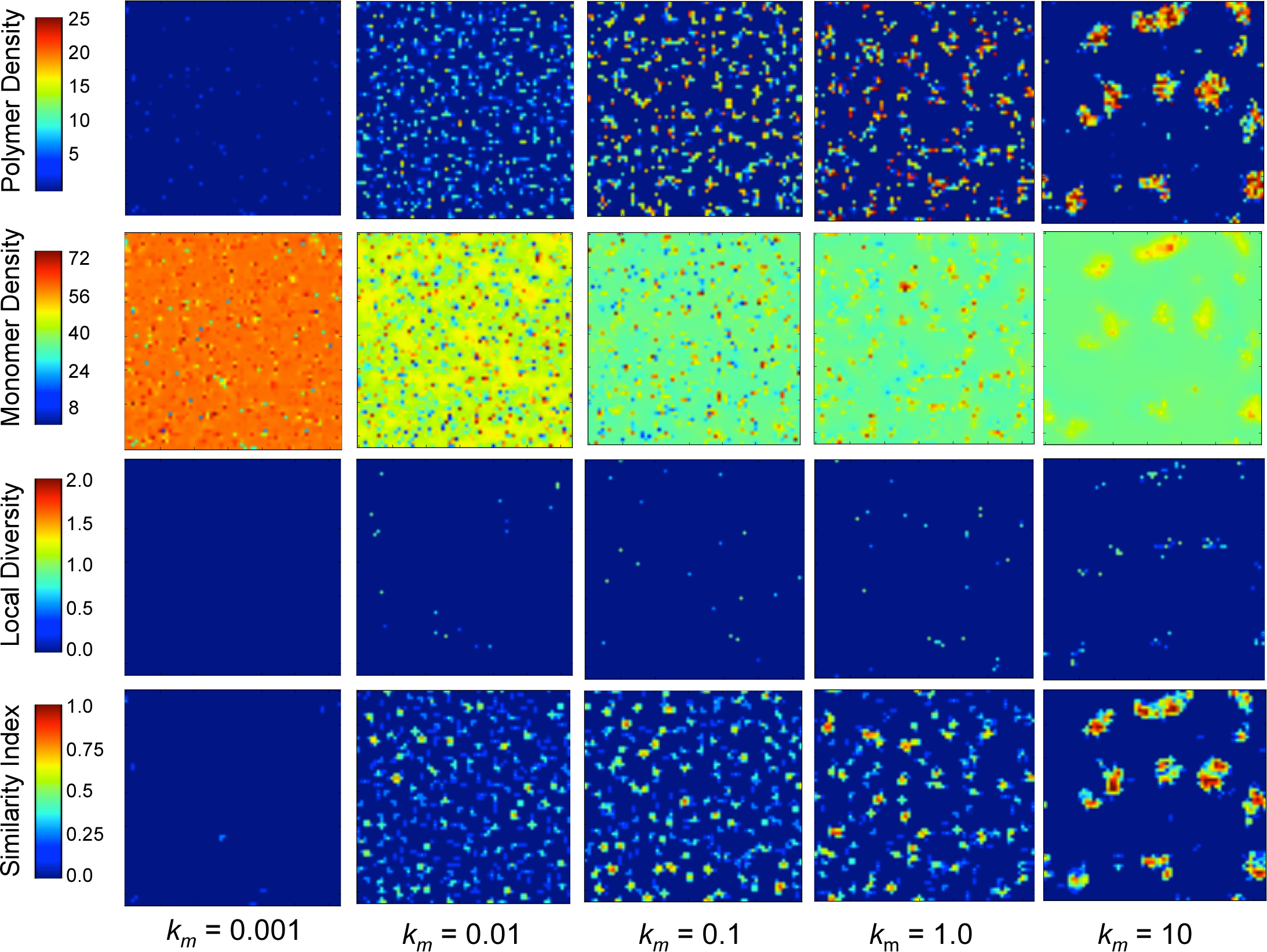

Supplement: Figure S2 — Spatial maps for sites/cycle. Spatial maps of polymer density (top row), monomer density (second row), local diversity (third row), and similarity index (bottom row) for sites/cycle, with monomer diffusivity increasing from left to right. Snapshots are taken at cycles. The kinetic rate constants are , , and . (TIF) [file pone.0034166.s002.tif]

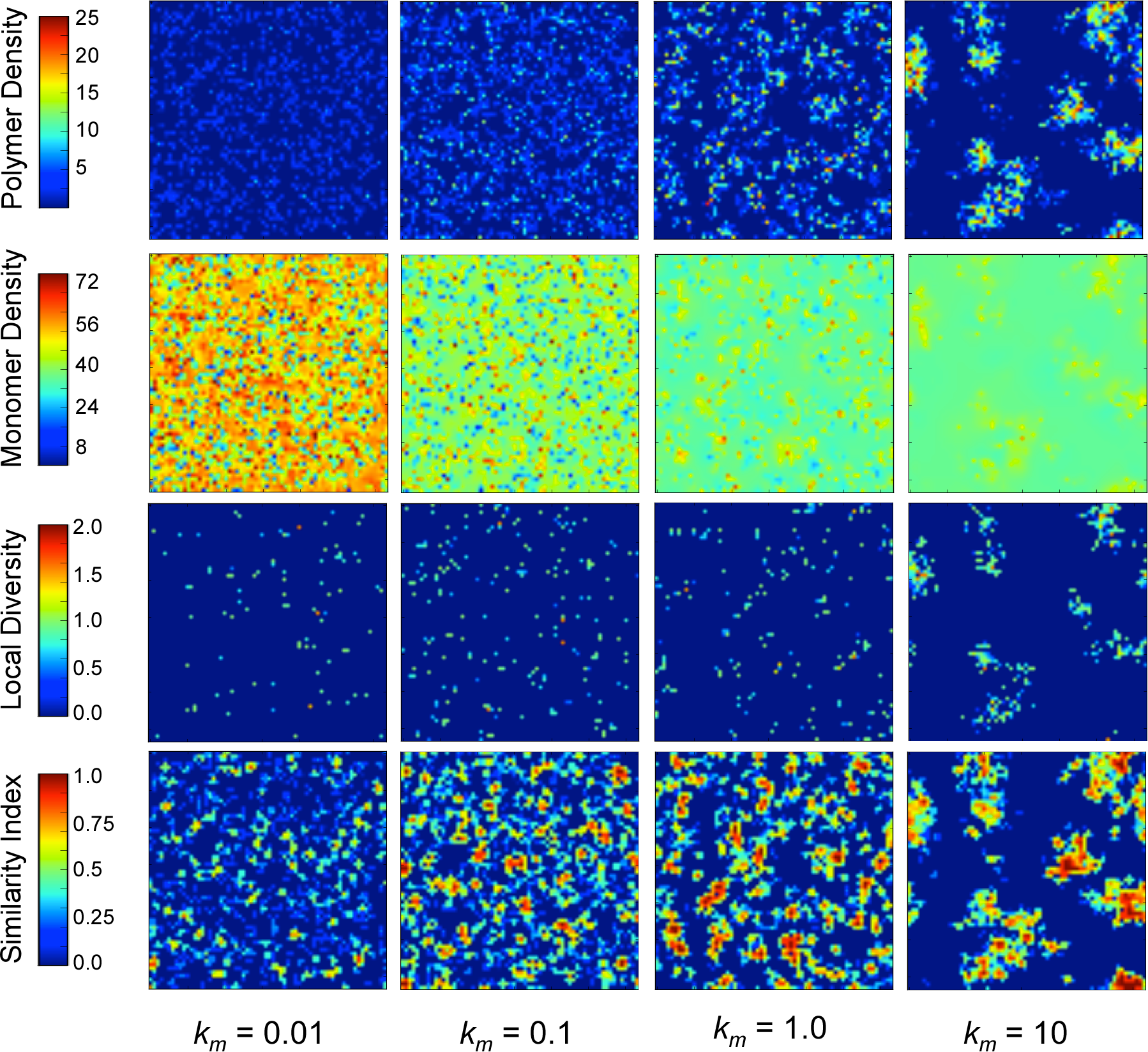

Supplement: Figure S3 — Spatial maps for sites/cycle. Spatial maps of polymer density (top row), monomer density (second row), local diversity (third row), and similarity index (bottom row) for sites/cycle, with monomer diffusivity increasing from left to right. Snapshots are taken at cycles. The kinetic rate constants are , , and . (TIF) [file pone.0034166.s003.tif]

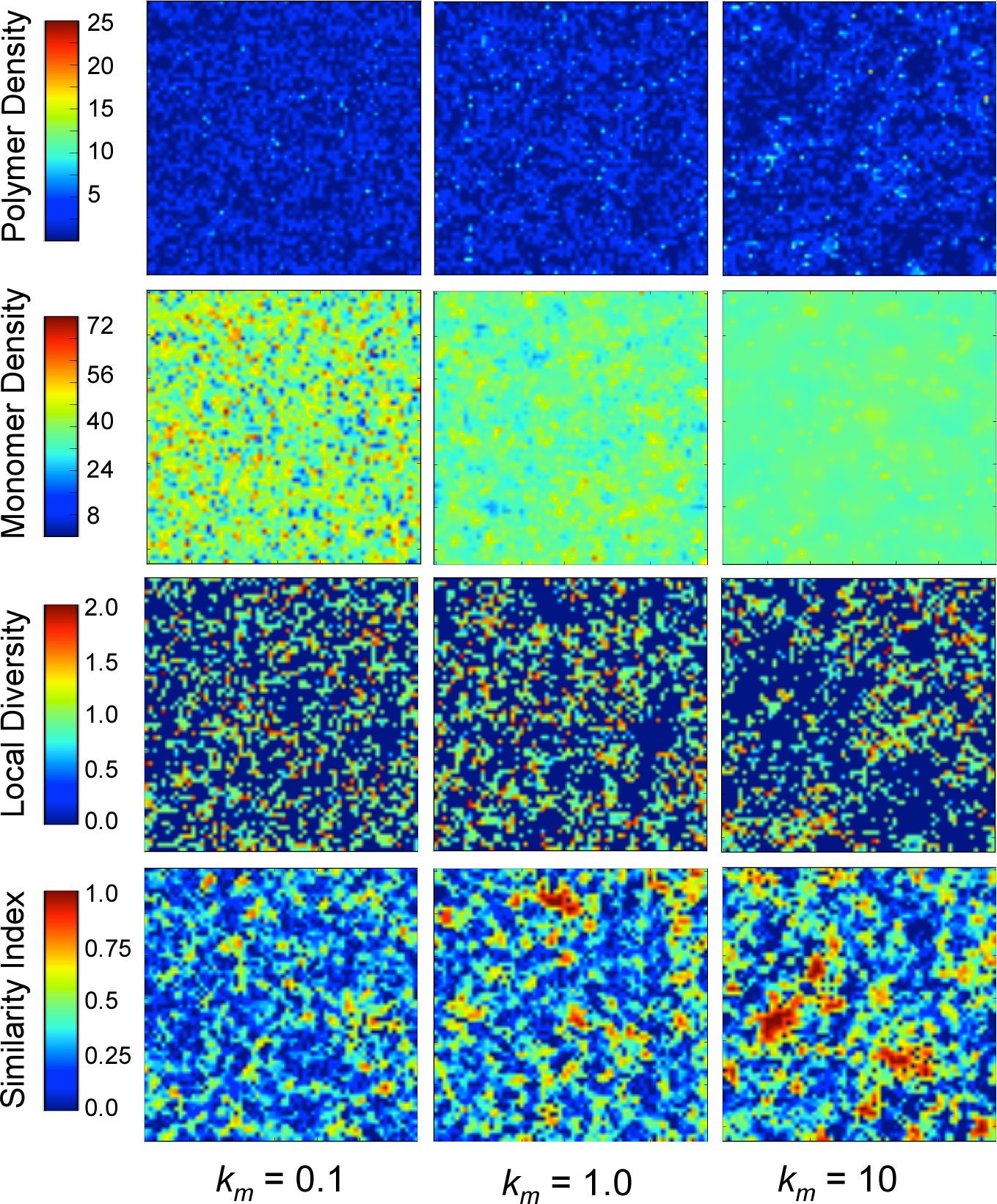

Supplement: Figure S4 — Spatial maps for sites/cycle. Spatial maps of polymer density (top row), monomer density (second row), local diversity (third row), and similarity index (bottom row) for sites/cycle, with monomer diffusivity increasing from left to right. Snapshots are taken at cycles. The kinetic rate constants are , , and . (TIF) [file pone.0034166.s004.tif]

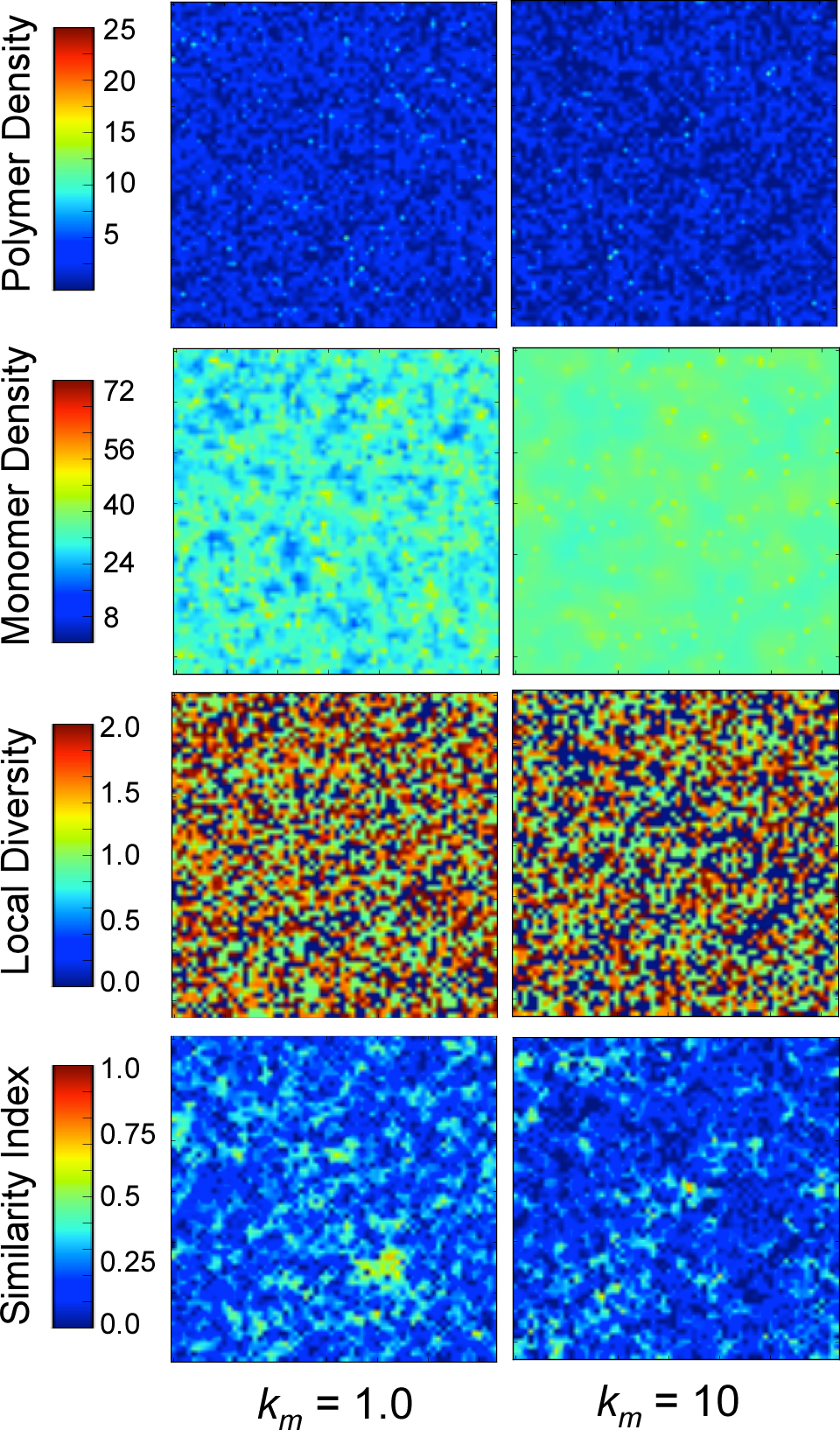

Supplement: Figure S5 — Spatial maps for sites/cycle. Spatial maps of polymer density (top), monomer density (second row), local diversity (third row), and similarity index (bottom row) for sites/cycle, with monomer diffusivity increasing from left to right. Snapshots are taken at cycles. The kinetic rate constants are , , and . (TIF) [file pone.0034166.s005.tif]

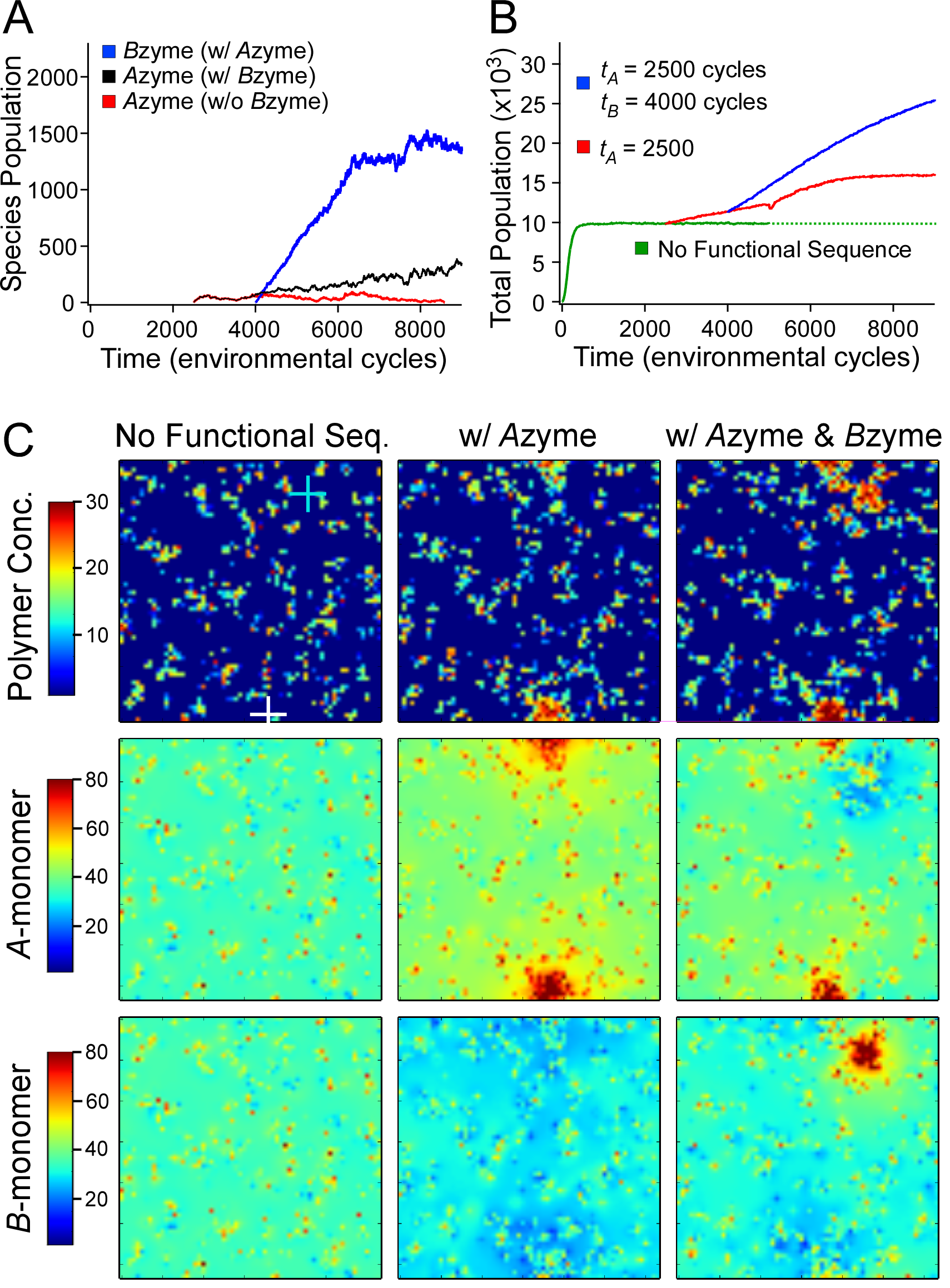

Supplement: Figure S6 — Spatial distribution maps for no functional species, one functional species, and two functional species. The three scenarios shown are all identical up to cycles, at which time the system has achieved a quasi-steady state distribution. In the first scenario, no functional sequences appear. In the second scenario, a functional zyme appears at = 2500. In the third scenario, the same functional zyme appears at = 2500 cycles, and the functional zyme appears at = 4000 cycles. In Panel A, the time evolution of the Species Populations of the zyme and zyme is shown. The red curve corresponds to the second scenario, having only the zyme, while the black and blue curves correspond to the third scenario with both enzymes emerging. Panel B shows the time evolution of the Total Polymer Population for the three scenarios. Panel C illustrates the spatial distribution of the polymer (total) and monomer concentrations, at = 5000 cycles. Kinetic rates are , , and , and diffusive rates of and sites/cycle. (TIF) [file pone.0034166.s006.tif]
