# Supplementary material for: Universal Sequence Replication, Reversible Polymerization and Early Functional Biopolymers: A Model for the Initiation of Prebiotic Sequence Evolution
Source: PLoS One. 2012 Apr 6;7(4):e34166. doi: 10.1371/journal.pone.0034166 (PMC3320909; doi:10.1371/journal.pone.0034166)
Supplement: Methods S1 — (PDF) [file pone.0034166.s009.pdf]

Supporting Information
Universal Sequence Replication, Reversible Polymerization and Early Functional Biopolymers: A Model for the Initiation of Prebiotic Sequence Evolution

Sara Imari Walker[1,2,3,4], Martha A. Grover[1,2] Nicholas V. Hud[1,3]

1 NSF/NASA Center for Chemical Evolution, Georgia Institute of Technology, Atlanta, GA, USA

2 School of Chemical and Biomolecular Engineering, Georgia Institute of Technology, Atlanta, GA, USA

3 School of Chemistry and Biochemistry, Georgia Institute of Technology, Atlanta, GA, USA

4 Current address: BEYOND: Center for Fundamental Concepts in Science, Arizona State University, Tempe, AZ, USA

1 Computational Methods

Simulations of the spatiotemporal dynamics of diffusion-limited informational polymers with environmentally driven assembly (both spontaneous and template-directed) and degradation were implemented via a hybrid kinetic Monte Carlo algorithm [1, 2], as outlined here.

1.1 Model Definition

Our model describing the dynamics of environmentally-driven recycling of informational polymers includes three kinetic and two diffusive processes:

1. Sequence-independent spontaneous assembly of polymers, with a rate constant k_s
2. Template-directed polymer replication via Universal Sequence Replication (USR), with a rate constant k_r
3. Sequence-independent polymer hydrolysis, with a rate constant k_h
4. Monomer diffusion, with hopping rate k_m (related to monomer diffusivity $\mathcal{D}_m$ by eq. 18)
5. Polymer diffusion, with hopping rate k_p (related to polymer diffusivity $\mathcal{D}_p$ by eq. 18).

The kinetic rate constants k_s, k_r, k_h, and diffusive hopping rate constants k_m and k_p are the tunable parameters in our model. Throughout we use italic and lowercase letters to denote dimensionless simulation values and upper-case letters to indicate the physical values of parameters. Since our aim is to study the influence of kinetic and physical parameters on system dynamics, additional model parameters, including total system mass and cycling rate, are held at a fixed values in this work. This simplified model permits us to explore a wide variety of chemistry-environmental couplings (*i.e.* a wide range of kinetic and diffusive parameter space) with the tractable set of just five model parameters.

The corresponding dimensionless mass-action kinetic equations for our model system are:

$$\frac{\partial A}{\partial t} = \mathcal{D}_m \nabla^2 A - R_{L_{1/2}} k_s AB - R_{L_{1/2}} k_r AB \sum_i X_i + R_{L_{1/2}} k_h \sum_i X_i \quad (1)$$

$$\frac{\partial B}{\partial t} = \mathcal{D}_m \nabla^2 B - R_{L_{1/2}} k_s AB - R_{L_{1/2}} k_r AB \sum_i X_i + R_{L_{1/2}} k_h \sum_i X_i \quad (2)$$

$$\frac{\partial X_i}{\partial t} = \mathcal{D}_p \nabla^2 X_i + k_s AB + k_r AB X_i - k_h X_i \quad (3)$$

where $R_{L_{1/2}}$ is half of the polymer length R_L (the motivation for including this factor is outlined below), and the sum over i runs over all extant sequences (*i.e.* all unique species in the population at time t). The variables A, B, and X_i correspond to the dimensionless concentrations of A monomer, B monomer, and polymer species with $ID = i$ respectively. This choice of kinetics is intended to approximate spontaneous assembly as a nucleation event, where the potential barrier for nucleation of a new sequence is high but once surmounted the sequence is easily assembled (see Section 2.2.4 in main text for more discussion). Therefore, the rate of spontaneous assembly is governed by the dimensionless second-order rate constant k_s and the local abundances $A(x, y)$ and $B(x, y)$. Likewise, replication is modeled as a third-order process (describing nucleation on a template), governed by a dimensionless sequence-independent third-order rate constant k_r, the local monomer concentrations $A(x, y)$ and $B(x, y)$, and the local abundance of polymer species i, X_i. Hydrolysis is governed by the first-order rate constant k_h and species abundance (see Section 2.2.4 in main text for more discussion). Length dependence is introduced to the kinetic equations through the factor $R_{L_{1/2}}$. The system has a conserved total mass, *i.e* we study a closed-mass system. Defining M as the total mass (the total number of monomeric units in monomer and as polymeric residues), we require $M = R_L \sum_i X_i + A + B$.

The number of possible polymer sequences N increases exponentially with the polymer length R_L; we therefore simplify our model by limiting the space of possible sequences through constraining the number of monomer species, the length of polymers, and the possible sequence content of polymers. We implement our model with two monomer species, labeled A and B. This choice is motivated by selecting the minimal system that will permit diversity of polymeric sequences. We consider only polymers with length $R_L = 20$, and each distinct polymeric sequence contains both 10 A-monomers and 10 B-monomers. This simplifies polymer identification: each unique species need only be identified by a single ID number, i, where the ID number contains enough information to fully identify the species. Our approximation to the full sequence space greatly simplifies the model while maintaining the essential dynamical features we wish to capture (see discussion in Section in 2.2.2 and 2.2.3 of the main text). It also justifies our implementation of the factor $R_{L_{1/2}}$ included in eqs. 1 - 3, because both monomer species A and B contribute $R_{L_{1/2}}$ residues to a given polymer. Additionally, the fixed polymer length of $R_L = 20$ was chosen to balance our requirements of having sufficient diversity in possible polymer sequences while keeping the dynamics numerically tractable. The number of possible polymers for $R_L = 20$ with a $50 : 50$, $A : B$ ratio is calculated by the binomial coefficient, $20!/(10!10!) = 184,756$. This set is large enough such that the simulations presented here only sample a small fraction (less than 5%) of the total number of possible sequences available in the potential sequence pool. The fixed value $R_L = 20$ can be taken to approximate a reaction-diffusion system supporting polymers with a mean-length of $\bar{L} = 20$ residues. These simplifications greatly alleviate the numerical requirements of our simulations, allowing us to run larger statistical samplings of our *in silico* experimental runs, while still permitting us to explore the most salient features of system dynamics. We have also explored systems supporting polymerization of shorter sequences, with $R_L < 20$, and observed that the dynamics are qualitatively similar, where the ratio $\frac{N_m}{N_p}$ scales with polymer length for a given set of kinetic parameters (N_m and N_p are the total number of monomers and polymers, respectively).

1.2 Model Implementation

The reaction surface is modeled on a 64×64 square lattice. The lattice size of 4096 sites was chosen to be small enough for numerical tractability, yet large enough to allow spatial correlations to be observed, for the ranges of kinetic and diffusive parameter under study. The initial conditions are homogeneous, with all mass in monomer: each of the 4096 lattice sites is initialized with 60 A and 60 B monomers. For functional runs each site is additionally initialized with 60 pA monomers. No polymers are present at the start of a given simulation run (with the exception functional sequence runs which start from an already established pool of random sequence polymers at quasi steady-state, see below). Periodic boundaries

were imposed to avoid edge-effects. However, additional simulations performed with closed boundaries revealed similar dynamics to those presented here. For example, in the case of simulation parameters leading to the emergence of localized clusters of high polymer density, we observe polymers preferentially aggregating at the corners of the square grid (not shown).

Each of the 4096 lattice sites on the two-dimensional grid represents a locally homogenous reaction domain, characterized by a locally interacting community of monomers and polymers of different sequences. All kinetic events occur locally (on-site). Interaction between lattice sites occurs through diffusive contact. Over the course of system evolution, large separations in polymer and monomer populations are observed, with a typical simulation ratios (at steady-state) of $\sim \frac{N_m}{N_p} = 300$, where N_m and N_p are the total numbers of monomers and polymers, respectively. To increase numerical efficiency, reaction and diffusion events are therefore partitioned such that relatively rare polymer reaction and diffusion events are treated stochastically and much more frequent monomer diffusion events are treated deterministically. This partitioning is implemented with a spatially explicit hybrid kinetic Monte Carlo algorithm [1,2] (see below). We have verified that the fully stochastic spatially-explicit kinetic Monte Carlo simulation [3] quantitatively reproduces the same dynamical phenomena (not shown).

The kinetic equations outlined in eqs. 1 − 3 do not explicitly account for environmental cycling. However, our numerical implementation via kinetic Monte Carlo simulations must explicitly take into account environmental cycling in order to accurately capture the desired dynamics. Our Monte Carlo implementation is therefore partitioned into two phases: a dehydrated phase, where all sites are diffusively isolated (*i.e.* diffusion is turned off); and a hydrated phase, where sites interact diffusively through polymer hopping events and monomer diffusion. The three kinetic processes are partitioned between these two phases such that polymer assembly and replication occur in the dehydrated phase, and polymer degradation and enzymatic catalysis (when functional sequences are extant) occur in the hydrated phase.

1.2.1 The Dehydrated Phase

Each lattice site x on the two-dimensional lattice is diffusively isolated and treated individually with the standard Gillespie algorithm [4]. The dehydrated phase supports two kinetic processes:

- Spontaneous assembly, with propensity:

$$a_s(x) = k_s A(x) B(x) \tag{4}$$

- Sequence-independent replication via template-directed assembly, with propensity:

$$a_{r_i}(x) = k_r A(x) B(x) N_i(x) \tag{5}$$

Here $A(x)$, $B(x)$, and $N_i(x)$ are the *number* of A monomers, B monomers, and polymer species i at site x (which is not strictly the same as the dimensionless concentrations cited for eqs. 1 − 3 above). The total propensity for polymer formation is

$$a_{tot}(x) = a_s(x) + \sum_i a_{r_i}(x) \ , \tag{6}$$

where the sum over i runs over all species indigenous to the site x. Events are drawn at random. Since the dynamics are environmentally driven, a polymer may copy itself *at most* once per hydration/dehydration cycle. Therefore, after each replication event the total number of polymers available for replication is decreased by one unit. As A and B monomers are incorporated into polymers over the course of the

dehydrated phase, the propensities for polymer formation through replication and spontaneous assembly decrease.

For all simulations presented in this work, the rate constant for spontaneous assembly is set to the dimensionless value $k_s = 10^{-7}$. This value corresponds to a maximal global nucleation rate of 1.47 new sequences per environmental cycle when no polymers are present (calculated by summing $a_s = k_s \times AB$ over all sites for the case where all mass is in monomer, *i.e.* $A = B = 60$ monomers per site for all 4096 lattice sites).

1.2.2 The Hydrated Phase

Diffusion and hydrolysis occur when the system is hydrated. In the hydrated-phase the dynamics are modeled with a spatially-explicit hybrid kinetic Monte Carlo algorithm [1, 2], where polymer diffusive hopping is treated as a stochastic event as in other spatially explicit treatments which incorporate diffusion in the Gillespie algorithm (see *e.g.* [3]). For the system presented here, a natural partition between rare and common events occurs due to the large separation in the population densities of monomer and polymer as discussed above. Our simulations are therefore hybridized such that monomer site hopping is coarse-grained by introducing the partial differential equation describing mass-action monomer diffusion:

$$\frac{\partial M(x)}{\partial t} = \mathcal{D}_m \nabla^2 M(x) \tag{7}$$

where $\mathcal{D}_m$ is the macroscopic monomer dimensionless diffusion rate (see eq. 14 below for relation to monomer hopping rate cited in the text), $M(x) = A(x)$ or $B(x)$ is the concentration of monomers per unit area, and ∇^2 is the two-dimensional Laplace operator. Eq. 7 is evolved forward in time using a standard finite-difference staggered leapfrog algorithm [5], with lattice spacing $dx = 0.2$ and time-step $dt = 0.01$. Implementation of this algorithm is subject to the stability criteria imposed by the Courant Condition yielding numeric stability for $\mathcal{D}_m < 1$, constraining our simulations to values $k_m < 100$ (see eq. 14), for the values of dx and dt implemented in the simulations.

Against the background of monomer diffusion, the rare polymer events of diffusion and degradation occur. These rare processes are:

- Polymer hydrolysis (degradation) with propensity

$$a_{h_i}(x) = k_h N_i(x) \tag{8}$$

- Polymer diffusion (hopping between nearest-neighbor lattice sites) with propensity

$$a_{p_i}(x) = k_p N_i(x) \tag{9}$$

The time-step between rare-events is calculated by determining the value of τ such that

$$\int_t^{t+\tau} a_{rare}(t')dt' = \ln\left(\frac{1}{\xi}\right) \tag{10}$$

where a_{rare} is the total propensity for rare reactions,

$$a_{rare} = \sum_x \sum_i a_{p_i}(x) + a_{h_i}(x) \ , \tag{11}$$

with events selected globally, and ξ is a random number drawn from the set $(0, 1]$ [1]. To calculate the integral in eq. 10, the numbers of all rare and common molecules must be followed in time. The

deterministic PDE of eq. 7 is used to track the evolution of the monomers species A and B. The PDEs are integrated forward in time using the staggered leapfrog algorithm until eq. 10 is satisfied. One stochastic event is then chosen to occur at random from the global pool of possible rare events on the lattice by a standard Gillespie algorithm [4], which is calculated only for rare events.

1.2.3 Functional Runs

In simulations including a functional Azyme, which catalyzes formation of A monomers from pA monomers, two additional processes are added to the hydrated phase: diffusion of pA monomers, and catalytic conversion of $pA \rightarrow A$. Diffusion of pA monomers is treated the same as $A-$ and $B-$ monomer diffusion using the mass-action monomer diffusion eq. 7. Catalysis is added to the rare events of the hydrated phase, with the probability of catalysis calculated from the reaction propensity:

$$a_{pA}(x) \quad = \quad k_c pA(x) N_j(x) \tag{12}$$

where $pA(x)$ is the dimensionless pA concentration (*i.e.* the number of pA monomers at site x), k_c is the microscopic catalysis rate for conversion of $pA \rightarrow A$ in the presence of the Azyme, and j is the ID number of the Azyme. The total propensity for rare events with an extant functional Azyme is therefore modified such that $a_{rare} = \sum_x (a_A(x) + \sum_i (a_{p_i}(x) + a_{h_i}(x))$.

As discussed in the main text, to illustrate the impact of the emergence of a functional sequence, data was saved at $t = 2500$ cycles for all details of a simulation, before continuing to run the simulation with no functional sequences present. This data provided the initial starting distribution of monomers and polymer species for the functional runs. The choice of starting at $t = 2500$ cycles is somewhat arbitrary, but was chosen to be sufficiently late in the system evolution that a quasi-steady state had been established (*i.e.* the ratio of monomer to polymer was relatively constant). To this initial condition, 60 pA monomers were added to each site (to model a previously untapped resource in the environment). A single polymer sequence representing the Azyme was randomly inserted on the lattice as a spontaneous assembly event (with the choice of site weighted by the propensities in eq. 4 for each run). Catalytic efficiency, k_c, was set to 100. Results were averaged over twenty-five runs (each with a randomly chosen insertion point for the inoculated sequence). The simulations were permitted to run until the inoculated sequence died out, or until the sequence had survived for 5000 cycles. The Bzyme was later inserted at $t = 4000$ cycles, catalyzing $pB \rightarrow B$.

2 Dimensionalization of Model Parameters

Throughout this work, we cite dimensionless microscopic kinetic and diffusive rates used in the kinetic Monte Carlo simulations. To recover dimensioned values we define the dimensionless time and space variables

$$t = \frac{T}{\gamma} \ , \qquad x = \frac{X}{\lambda} \ , \tag{13}$$

where γ is a typical time scale, and $\lambda = \sqrt{D\gamma}$ is a typical spatial scale, and D is a dimensioned diffusion rate which we scale out of the equations such that:

$$\mathcal{D}_m = \frac{D_m}{D} \ , \qquad \mathcal{D}_p = \frac{D_p}{D} \tag{14}$$

The system is subject to the physical constraint $\frac{D_p}{D_m} \geq 1$. With this parameterization, the dimensionless kinetic rate constants implemented in our simulations (indicated by lower-case letters) are related to their physical counterparts (denoted by upper-case letters) as:

$$k_h = \gamma K_h \ \ , \ \ k_s = \frac{\gamma K_s}{\lambda^2} \ \ , \ \ k_A = \frac{\gamma K_A}{\lambda^2} \ \ , \ \ k_r = \frac{\gamma K_r}{\lambda^4} \tag{15}$$

for hydrolysis (first-order process), spontaneous assembly and enzymatic catalysis (second-order processes), and templated-directed replication (third-order process), respectively. Dimensionless concentrations may be made dimensional by the transformation:

$$A = [A]\lambda^2 \ , \qquad B = [B]\lambda^2 \ , \qquad N_i = [X_i]\lambda^2 \tag{16}$$

where $[A]$ is the concentration of A monomers, $[B]$ is the concentration of B monomers, and $[X_i]$ is the concentration of polymer species with ID number i. The notation $[\ldots]$ is used to indicate concentration in number of molecules per unit area such that the variables A, B, and N_i correspond to the number of molecules within a spatial region of size λ^2. As shown below, it is convenient to take the length scale λ to be the physical size of a lattice site.

To model diffusion events on the lattice, a relation between the mass-action diffusivities defined above, and the microscopic hopping rates must be defined. The microscopic diffusive hopping rate for polymers is defined as:

$$k_p = \frac{4\mathcal{D}_p}{dx^2} \tag{17}$$

where we have used the mean-square displacement for particles subject to Brownian diffusion $\langle r^2 \rangle = 2d\mathcal{D}_p\tau$, with dimensionality $d = 2$, and a mean displacement $\langle r^2 \rangle = dx^2$, noting that $\tau = k_p^{-1}$ is the hopping time between sites. It is the dimensionless hopping rate k_p which we use as a simulation parameter and therefore cite throughout this work. The dimensional diffusivity D_p can be recovered using eqs. 14 and 17.

Since monomer diffusion is treated deterministically via a mass-action diffusion equation, the monomer diffusion parameter used in our simulations is $\mathcal{D}_m$. However, we additionally define a dimensionless monomer hopping rate:

$$k_m = \frac{4\mathcal{D}_m}{dx^2} \tag{18}$$

which is calculated in an analogous manner to k_p. Throughout this work we cite the dimensionless hopping rate k_m, rather than the simulation parameter $\mathcal{D}_m$, to provide an more direct comparison between monomer and polymer diffusion rates and timescales.

For numerical simplicity, we normalize the physical size of a lattice site L, to $L^2 = 1\ l^2$, where l is a unit of length (*e.g.* $l = \mu$m, mm, or cm), such that the number of molecules on a lattice site is equal to its dimensionless concentration (*i.e.* $N_i = X_i$ where X_i is the dimensionless concentration of species i cited in eqs. 1 – 3). Consequently, the non-dimensional size of a lattice site is $dx = 0.2$ for our simulations, and using eq. 13 this yields a constraint condition

$$\sqrt{D\gamma} = 5\ l, \tag{19}$$

(where we have used $\lambda = \sqrt{D\gamma} = \frac{L}{dx}$), in our model parameterization. Scaling parameters D and γ must be chosen to satisfy eq. 19, to be consistent with our numerical implementation. Because it is easiest to define a timescale γ relative to the typical physical length of an environmental cycle, this usually translates to a constraint on the scaling parameter D, which is otherwise an arbitrary parameter. This choice therefore does not affect interpretation of our results, and enables straightforward recovery of dimensionalized values for simulation parameters by defining typical length and timescales λ and γ only.

3 Data Analysis

In the absence of functionality, the system comes to a quasi steady-state of dynamic kinetic equilibrium after several hundred cycles. The exact timescale depends on the particular diffusive and kinetic rates

of a given system. Equilibrium is defined by a quasi steady-state ratio of monomer to polymer. That is, $\frac{d(N_m/N_p)}{dt} \simeq 0$, where N_m is the total number of monomers and N_p is the total number of polymers, with stochastic fluctuations about the equilibrium value. The quasi steady-state distribution is dynamic, with the population of extant polymers continuously changing with time. Nonetheless, certain global measurements, including the total number of polymers, the global and averaged local diversity (defined below), the average number of extant species, and the average extant species population size and lifetime, reach values during the quasi steady-state that are characteristic of a given set of system parameters (*i.e.* these values are deterministic, with stochastic fluctuations about a characteristic value). To make comparisons between different systems, with different sets of kinetic and diffusive parameters, we time-averaged these characteristic values during the quasi-steady state system evolution. Data is time-averaged over 2500 cycles and then ensemble averaged over a small statistical sampling of runs (for work presented here these sample sizes are 5 or 10 runs). Small statistical samples are sufficient given the small spread in simulation values and the length of simulations with time-sampling over 2500 cycles. The quasi-steady state distribution values are calculated starting at $t = 2500$ cycles, to ensure that all systems have achieved a steady-state distribution of monomer to polymer for all parameter ranges explored (typically steady-state is achieved at $t = 500 - 1000$ cycles depending on simulation parameters). Error bars correspond to sample standard deviation on the mean time-averaged values.

For runs illustrating the emergence of a functional sequence, where a random sequence is inoculated at a randomized location on the lattice (weighted by the propensities for spontaneous assembly at each site), lifetimes are averaged over survival times for the inoculated sequence lineage taken over all twenty-five experimental runs. Population size averages, as well as the average number of extant species and exploration rate, are averaged over the sequence lifetime (*i.e.* for a polymer that lives 5 cycles this corresponds to an average over 5 cycles) and therefore yield much higher variances in the data set than for the nonfunctional simulations which are averaged over entire populations of thousands of sequences, over thousands of environmental cycles.

3.1 Local Diversity

Each lattice site is characterized by its sequence population. To calculate the local (on-site) diversity of polymer populations we use the Shannon equation for information [6], used in studies of species diversity (see *e.g.* [7]). Local diversity is calculated for each site individually as,

$$S_L(x,t) = -\sum_i \frac{N_i(x,t)}{N_{tot}(x,t)} \ln\left(\frac{N_i(x,t)}{N_{tot}(x,t)}\right) \tag{20}$$

where i sums over all unique polymer species on the local site x, $N_i(x,t)$ denotes the local population size of each unique polymer species i on site x at time t, and N_{tot} is the total number of polymers on the local site x at time t (*i.e.* $N_{tot}(x,t) = \sum_i N_i(x,t)$). Local diversity measures the statistical diversity of each lattice site and is spatially averaged over all sites x to yield the average local diversity

$$\langle S_L(t) \rangle = \frac{\sum_x S_L(x,t)}{4096} \tag{21}$$

where 4096 is the total number of local populations (lattice sites) on our 64×64 square lattice. It is this spatially-averaged local diversity that is reported in our ensemble averaged data analysis (with spatial maps of local diversity included for simulation snapshots). Average local diversity therefore provides a statistical measure of the extent to which multiple sequences coexist on the same lattice site. As such, it provides a measure of diffusive mixing of populations and competition, whereby spatial regions with low local diversity result either from low diffusivity or high rates of local resource competition that result in fewer unique species.

3.2 Similarity Index

As an added measure of the spatial diversity of the polymer population we utilize a generalization of the of the Jaccard similarity index used in population ecology [7, 8]. This index provides a statistical measure of the similarity between next-nearest neighbor "communities" or lattice sites. Adopting this concept to chemical evolution, the similarity between two lattice sites α and β containing N_α and N_β unique sequences, respectively, and sharing $N_{\alpha\beta}$ mutual sequences is given by:

$$I_{\alpha\beta}(t) = \frac{R_\alpha(t)R_\beta(t)}{R_\alpha(t) + R_\beta(t) - R_\alpha(t)R_\beta(t)} \tag{22}$$

where R_α is the sum of the relative abundances of the shared species at site α:

$$R_\alpha(t) \equiv \sum_i^{N_{\alpha\beta}} \frac{N_i(\alpha,t)_{shared}}{N_{tot}(\alpha,t)} \ . \tag{23}$$

Here $N_i(\alpha,t)_{shared}$ is the abundance of the i-th species shared between sites α and β that are on site α at time t, and N_{tot} is the sum total of all polymers of both shared and unshared species at site α (*i.e.* $N_{tot}(\alpha,t) = \sum_i N_i(\alpha,t)$). An equivalent definitions for R_β is obtained with the substitution $\alpha \leftrightarrow \beta$.

A map of similarity of nearest-neighbor sites is produced by calculating the pairwise similarity index $I_{\alpha j}$ at the site α, where the index j runs over the eight nearest neighbor communities $j = 1, 2, ...8$. For each site, the indices $I_{\alpha j}$ are averaged to yield the site specific value:

$$I(x_\alpha, y_\alpha) = \frac{\sum_j I_{\alpha j}}{8} \qquad j = 1, 2, ...8 \tag{24}$$

The value $I(x_\alpha, y_\alpha)$ is recorded as the similarity index for site α in the similarity map. This procedure provides a method of identifying regions in the system occupied by highly similar populations of polymers as well as aiding in visual identification of clustered regions as shown in the Supporting Figures below.

3.3 Sequence Space Exploration Rate, Extant Species, and Species Lifetime

The total number of species introduced to the system, $N_S(t)$, is tracked over the entire course of each simulation and provides a measure of the total size of sequence space explored as a function of time. The rate of exploration of sequence space $R_S(t)$, *i.e.* the species production rate, is therefore calculated as the slope of the time-dependent total species, *e.g.* $R_S(t) = \dot{N}_S(t)$, discretized as $R_S(t_i) = \frac{N_S(t_i) - N_S(t_{i-1})}{2}$, which achieves a constant (within small fluctuations) and nonzero value during the quasi steady-state system evolution. $R_S(t)$ is time-averaged to obtain a mean exploration rate for each simulation run. Since the total number of extant species, N_{ES} (a directly measured value) achieves a constant value (within small fluctuations) during the quasi steady-state evolution, the number of species created per unit time is equal to the number destroyed as the extant population explores sequence space. An estimate of the mean species lifetime τ_S is therefore calculated as $\tau_S = N_{ES}/R_S$.

References

1. Alfonsi A, Cancès E, Turinici G, Di Ventura B, Huisinga W (2004) Exact simulation of hybrid stochastic and deterministic models for biochemical systems. Rapport de recherche RR-5435, INRIA.

2. Chatterjee A, Vlachos DG (2007) An overview of spatial microscopic and accelerated kinetic Monte Carlo methods. J Comput-Aided Mater 14: 253–308.

3. Bernstein D (2005) Simulating mesoscopic reaction-diffusion systems using the Gillespie algorithm. Phys Rev E 71: 41103.

4. T D, Gillespie (1976) A general method for numerically simulating the stochastic time evolution of coupled chemical reactions. J Comput Phys 22: 403–434.

5. Press W, Teukolsky S, Vetterling W, Flannery B (1992) Numerical Recipes in C: The Art of Scientific Computing. Cambridge University Press, 2nd edition.

6. Shannon C (1948) A mathematical theory of communication. Bell Syst Tech J 27: 623–656.

7. Filotas E, Grant M, Parrott L, Arne P (2010) Positive interactions and the emergence of community structure in metacommunities. J Theor Biol 266: 419–429.

8. Chao A, Chazdon RL, Colwell RK, Tsung-Jen S (2005) A new statistical approach for assessing similarity of species composition with incidence and abundance data. Ecol Lett 8: 148–159.
