# Supplementary material for: Universal Sequence Replication, Reversible Polymerization and Early Functional Biopolymers: A Model for the Initiation of Prebiotic Sequence Evolution
Source: PLoS One. 2012 Apr 6;7(4):e34166. doi: 10.1371/journal.pone.0034166 (PMC3320909; doi:10.1371/journal.pone.0034166)
Supplement: Table S1 — Parameters and values used in simulations. (PDF) [file pone.0034166.s010.pdf]

Parameter	Description	Simulation Value(s)
N	Lattice size – the simulation surface is defined as an $N \times N$ grid	64
dx	Spatial discretization for calculating deterministic monomer diffusion	0.2
dt	Time-step for calculating deterministic monomer diffusion	0.01
M_N	Initial number of *each* monomer species on each lattice site at the start of a simulation run	60
M_{nf}	Total simulation mass (in monomeric units) for nonfunctional simulations	4.9042×10^5
M_f	Total simulation mass (in monomeric units) for functional simulations	7.3728×10^5, 9.8304×10^5
t_h	Length of the hydrated phase (in units of dimensionless time)	1.0
t_d	Length of the dehydrated phase (in units of dimensionless time)	1.0
N_L	Length of polymers (this fixed length is meant to approximate the mean of a distribution of polymer lengths)	20
k_s	Rate constant for spontaneous assembly of random sequence polymers from monomer during the dehydrated phase	10^{-7}
k_r	Rate constant for template-directed sequence-independent replication during the dehydrated phase	0, 10^{-5}, 10^{-4}, 10^{-5}
k_h	Rate constant for spontaneous polymer degradation during the hydrated phase	10^{-5}, 10^{-4}, 10^{-3}, 0.01, 0.1, 0.5, 1.0
k_m	Hopping rate of monomers between lattice sites during the hydrated phase	0, 0.001, 0.005, 0.01, 0.05, 0.1, 0.5, 1.0, 5.0, 10.0, 30.0, 50.0, 70.0, 90.0
k_p	Hopping rate of polymers between lattice sites during the hydrated phase	0, 0.001, 0.01, 0.1, 1.0
k_c	Rate constant for catalyzed monomer synthesis in the presence of a functional polymer with monomer synthetase activity	100
